# Network analysis identifies strain-dependent response to tau and tau seeding-associated genes

**DOI:** 10.1084/jem.20230180

**Published:** 2023-08-22

**Authors:** Dominic J. Acri, Yanwen You, Mason D. Tate, Hande Karahan, Pablo Martinez, Brianne McCord, A. Daniel Sharify, Sutha John, Byungwook Kim, Luke C. Dabin, Stéphanie Philtjens, H.R. Sagara Wijeratne, Tyler J. McCray, Daniel C. Smith, Stephanie J. Bissel, Bruce T. Lamb, Cristian A. Lasagna-Reeves, Jungsu Kim

**Affiliations:** 1 Stark Neurosciences Research Institute, Indiana University School of Medicine, Indianapolis, IN, USA; 2 Medical Neuroscience Graduate Program, Indiana University School of Medicine, Indianapolis, IN, USA; 3 Department of Anatomy, Cell Biology and Physiology, Indiana University School of Medicine, Indianapolis, IN, USA; 4 Department of Medical and Molecular Genetics, Indiana University School of Medicine, Indianapolis, IN, USA; 5 Department of Biochemistry and Molecular Biology, Indiana University School of Medicine, Indianapolis, IN, USA; 6 Center for Computational Biology and Bioinformatics, Indiana University School of Medicine, Indianapolis, IN, USA

## Abstract

Previous research demonstrated that genetic heterogeneity is a critical factor in modeling amyloid accumulation and other Alzheimer’s disease phenotypes. However, it is unknown what mechanisms underlie these effects of genetic background on modeling tau aggregate-driven pathogenicity. In this study, we induced tau aggregation in wild-derived mice by expressing *MAPT*. To investigate the effect of genetic background on the action of tau aggregates, we performed RNA sequencing with brains of C57BL/6J, CAST/EiJ, PWK/PhJ, and WSB/EiJ mice (*n* = 64) and determined core transcriptional signature conserved in all genetic backgrounds and signature unique to wild-derived backgrounds. By measuring tau seeding activity using the cortex, we identified 19 key genes associated with tau seeding and amyloid response. Interestingly, microglial pathways were strongly associated with tau seeding activity in CAST/EiJ and PWK/PhJ backgrounds. Collectively, our study demonstrates that mouse genetic context affects tau-mediated alteration of transcriptome and tau seeding. The gene modules associated with tau seeding provide an important resource to better model tauopathy.

## Teaser

Acri and colleagues report transcriptomic responses to tau and elevated tau seeding activity in wild-derived mice relative to C57BL/6J genetic background. This paper creates a rich resource by combining genetics, tau biosensor assays, and transcriptomics.

## Graphical Abstract

**Figure:**
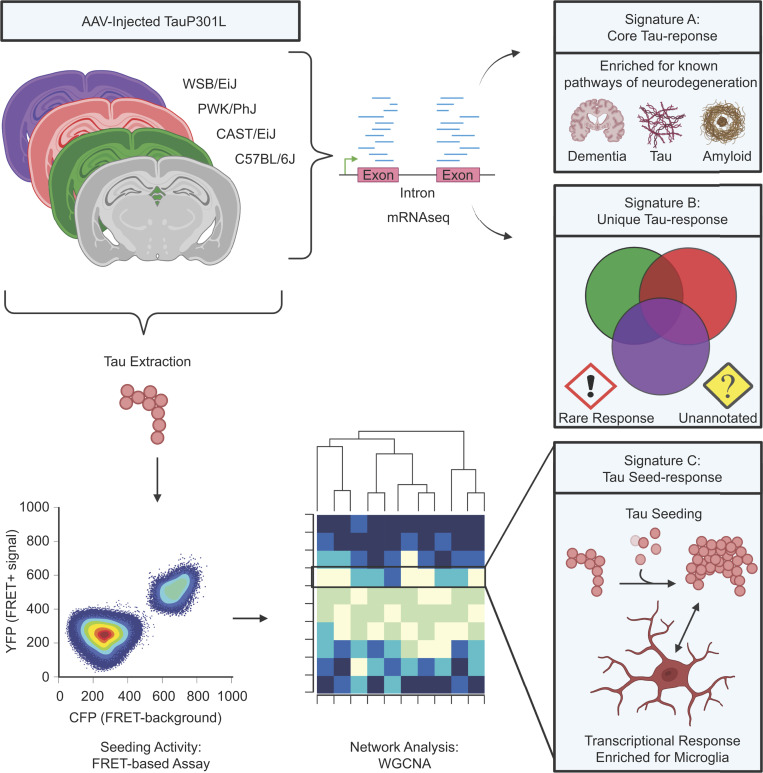

## Introduction

Alzheimer’s disease (AD) is the most common cause of dementia and is characterized by the accumulation of amyloid plaques and neurofibrillary tangles mainly comprised of aggregated tau protein (Long and Holtzman, 2019). Human genetic studies have identified variants that implicate several risk genes that influence AD pathogenesis (Karch and Goate, 2015). As researchers design studies to investigate the role of these late-onset AD risk genes, they must first decide which pathological outcome(s) to measure. A combination of transgenic, viral, and xenograft approaches have been developed to study amyloid-only, tau-only, and amyloid-tau pathogenesis in mice. While the ultimate goal of these studies is to translate findings to patients with AD, the first step to translation is understanding what is fundamentally happening in the model organism.

There are a number of promising therapeutic approaches that target tau (Congdon and Sigurdsson, 2018). Importantly, hyperphosphorylated tau has been shown to cause neuronal cell death (Lee et al., 2011) and to correlate with measures of cognitive decline (Arriagada et al., 1992) in humans. To investigate the progression of tauopathy, the most widely used mouse models express microtubule-associated protein tau (*MAPT*), the gene which encodes the tau protein. The P301L mutation originally described in frontotemporal dementia patients (Hutton et al., 1998; Poorkaj et al., 1998) is often used to induce tau aggregate formation and study tau pathogenesis. Transgenic models of Tau^P301L/S^ (Santacruz et al., 2005; Yoshiyama et al., 2007) and viral models of Tau^P301L^ (Cook et al., 2015; Wegmann et al., 2017) are useful tools to study the progression of tau pathology and investigate factors that could lead to the risk of developing any tauopathy, including AD. These models have been shown to recapitulate key aspects of human tauopathy including behavioral deficits (Lasagna-Reeves et al., 2016; Cook et al., 2014), neuroinflammation (Yoshiyama et al., 2007), prion-like proteopathic seeding (Martinez et al., 2022), and propagation from one cell to another (Dujardin et al., 2022; Rauch et al., 2020; Wegmann et al., 2017; de Calignon et al., 2012; Woerman et al., 2017).

Although the exact mechanism by which tau aggregates form is currently unknown, there is strong evidence for the role of “tau seeding” as an initiating event. Proteopathic tau seeds are capable of entering a cell and promoting aggregation in a prion-like manner (Clavaguera et al., 2009; Frost et al., 2009). Several studies have shown that seeding precedes tau pathogenesis and can even occur in brain regions where tau pathology does not usually present (DeVos et al., 2018; Kaufman et al., 2018; Stopschinski et al., 2021). Several in vitro models have been developed that can measure tau seeding activity from human patients or mouse models of tauopathy (Bengoa-Vergniory et al., 2021; Holmes et al., 2014; Jin et al., 2022). These seeding activity assays have assisted in the discovery of novel tau interactors and been used to investigate phosphorylation patterns associated with tau progression (Martinez et al., 2022; Mirbaha et al., 2022). Unlike human patients who are genetically diverse, most studies use the same monogenic mouse models. Therefore, the influence of genetic diversity on tau pathology and seeding has not been thoroughly investigated. With the hope that these preclinical studies will translate to tau-targeted treatments, there is a need to better understand how the genetic context of our mouse models affects our interpretation of tauopathy.

The most widely used mouse strain in biomedical research, the C57BL/6J strain (herein referred to as B6), was established by the Jackson Laboratory in the 1920s and became the strain used to create the mouse reference genome (Mekada et al., 2009; Mouse Genome Sequencing et al., 2002). While one goal of sustaining a single inbred line is to limit interlaboratory artifacts, research on B6 mice reveals that genetic drift and mixed background breeding have introduced a number of variants since the first draft of the mouse reference genome (Sarsani et al., 2019; Simon et al., 2013). These variants and others purposefully introduced by selective breeding are termed “genetic diversity.” Unique phenotypes arising from mouse genetic diversity can be used as a model for complex diseases. For example, decreases in pancreatic insulin at 12 wk of age in the NOD/ShiLtJ mouse model established this strain as the leading model for research in type 1 diabetes (Makino et al., 1980). Another key strategy in harnessing mouse genetic diversity is to breed together different mouse strains to create multiparent panels for genetic mapping (Churchill et al., 2004, 2012; Peirce et al., 2004).

The founder strains of the Jackson Laboratory’s multiparent panels, the Diversity Outbred and Collaborative Cross mice, include five classically inbred and three wild-derived mouse strains (Churchill et al., 2012). These eight founders were selected as they could be bred together to contain segregating variants every 100–200 base pairs (Churchill et al., 2004). The most genetically distinct of the eight founder strains are the wild-derived: CAST/EiJ, PWK/PhJ, and WSB/EiJ (herein referred to as CAST, PWK, and WSB). These three wild-derived strains are descendants of three different subspecies of *Mus musculus* and contain millions of variants relative to the mouse reference genome (Yang et al., 2011). For this reason, wild-derived mouse strains have been used as a resource for modeling the population-level heterogeneity that cannot be investigated using classical inbred mouse strains alone. Deep characterization of these wild-derived mice has uncovered genetic (Morgan et al., 2015), behavioral (Kollmus et al., 2020), and immune (Lilue et al., 2018) differences that are improving our knowledge of mouse genetics.

Previous research has demonstrated the importance of studying these wild-derived mice in the context of AD. Mice with APP^swe^ and PSEN1^de9^ transgenes (*APP/PS1* transgenic: B6.Cg-Tg(Appswe,PSEN1dE9)85Dbo/Mmjax) were backcrossed onto each of these three wild-derived mouse backgrounds. These mice had higher levels of amyloid-β (Aβ) compared with age-matched mice on a B6 background (Onos et al., 2019). Notably, Onos and colleagues observed an increase in neuroinflammation in the cortex and hippocampus of PWK mice. This is especially interesting given the important role of neuroinflammation in Aβ accumulation in AD (Efthymiou and Goate, 2017; Karahan et al., 2021; Mhatre et al., 2015; Schoch et al., 2021). Single-cell RNA sequencing (RNA-seq) of sorted microglia further demonstrated that the responses of immune cell subtypes, namely homeostatic microglia and disease-associated microglia (DAMs), are determined by wild-derived genetic backgrounds (Yang et al., 2021). As the increasing focus is spent on defining microglial subtypes in studies of neurodegeneration (Keren-Shaul et al., 2017; Paolicelli et al., 2022), the effect of wild-derived genetic background could be an important factor in selecting a mouse model that better reflects human disease. Even though wild-derived backgrounds have been shown to have a large effect on modeling Aβ pathology, little is known about their effect on modeling tauopathy.

Given the known effect of wild-derived mice on modeling Aβ accumulation, we aimed to investigate the effect of wild-derived mouse genetic background on tauopathy. To preserve the mouse genetic background, we expressed mutant tau with the P301L mutation in the brains of B6, CAST, PWK, and WSB mice using intracerebroventricular injection of an adeno-associated virus (AAV; Carlomagno et al., 2019; Cook et al., 2015). This strategy allows us to change the genetic background without the need to backcross a conventional transgenic mouse with each strain for 10+ generations. In addition to saving time and money using our viral approach, most importantly, we ensure that each genetic background is preserved without any possible genetic drift or the addition of unwanted variants over a multiyear backcrossing experiment. We found that the presence of seed-competent tau was modulated by genetic background, independent of human tau expression level. Using bulk mRNA-seq, we report transcriptional changes that are shared across genetic backgrounds, changes that are unique to wild-derived mice, and changes that are associated with the presence of seed-competent tau (Signatures A, B, and C, respectively). Our data serve as a resource for those studying the pathogenesis of tau and implicate several transcriptional signatures that are not present when modeling tauopathy in B6 mice.

## Results

### Variants in wild-derived genetic backgrounds within Accelerating Medicines Partnership Program for AD (AMP-AD) nominated target genes

**Figure 1.**
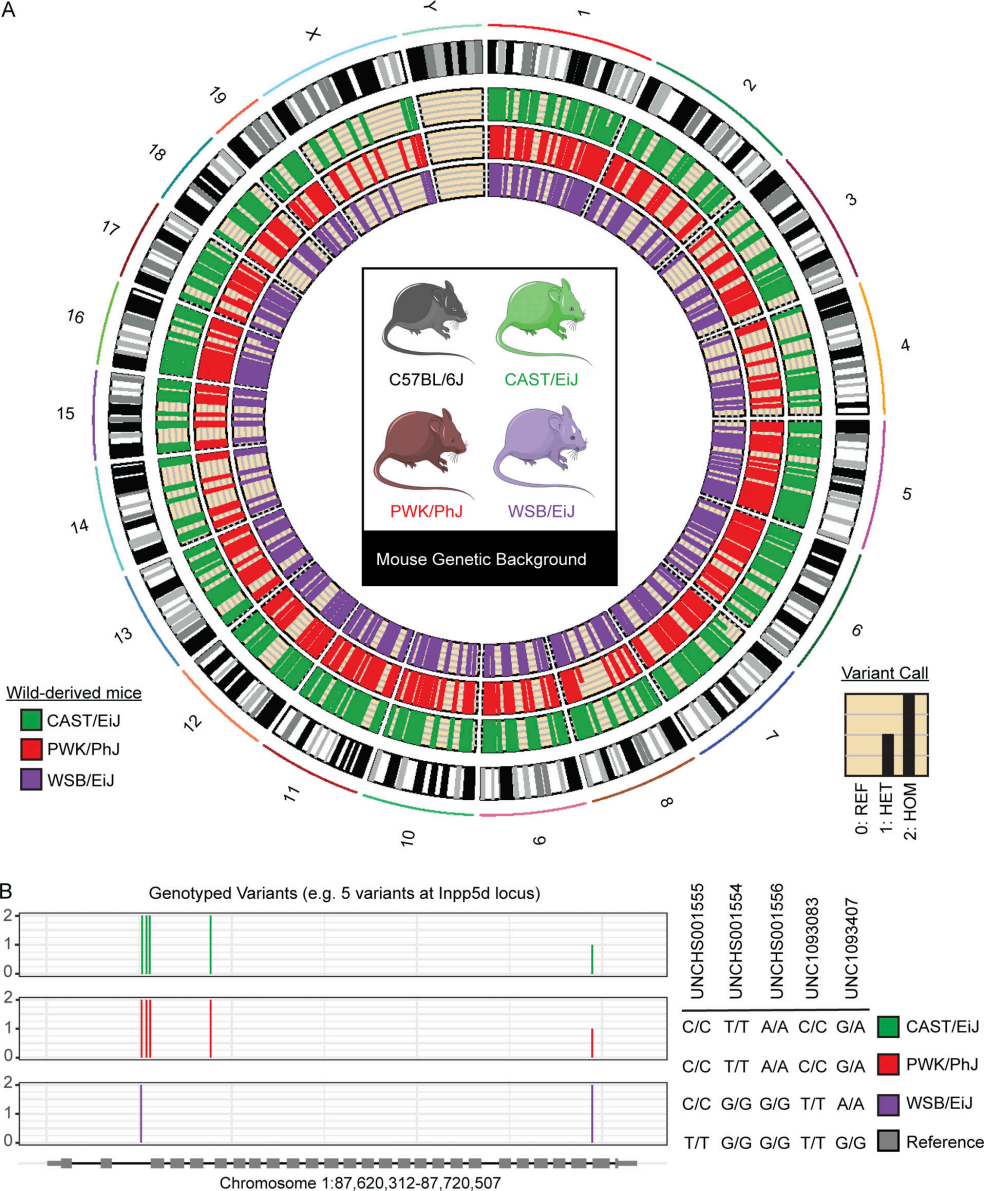
**Variants in wild-derived genetic backgrounds within AMP-AD nominated target genes.** Classical inbred mouse model C57BL/6J and three wild-derived mouse genetic backgrounds (CAST, PWK, and WSB). **(A)** Variants in wild-derived mice were called using gigaMUGA relative to the reference genome (C57BL/6J) and recorded (0: reference call, 1: heterozygous variant, 2: homozygous variant) using the GenCall Algorithm implemented in the Illumina BeadStudio software per manufacturer’s recommendations. Wild-derived mice contain 5,810 variants in the 537 nominated target genes from the AMP-AD consortium (accessed March 1, 2021). **(B)** Five genotyped variants at the Inpp5d locus (Chr1:87,620,312–87,720,507) demonstrate genetic heterogeneity within a single known AD risk gene.

CAST, PWK, and WSB mice contain millions of variants across the mouse genome (Blake et al., 2021). These variants include over 5 million single nucleotide polymorphisms (SNPs; Onos et al., 2019), 116 novel genes not present in B6 (Lilue et al., 2018), and between 250 and 400 large structural variants (Yalcin et al., 2011). To identify genetic variants in our mice, we used the Illumina Infinium platform, containing 143,259 probes, designed specifically for wild-derived mice and other founders of the Diversity Outbred mouse model (Morgan et al., 2015). This allowed us to confirm the genotype of each genetic background in our laboratory and gave us information about the SNPs and copy number variants in structurally polymorphic regions of the mouse genome. To determine which of these variants could be important in AD research, we focused on target genes nominated by the AMP-AD consortium. The genotyped variants of CAST, PWK, and WSB within the AMP-AD nominated targets were visualized using a circos plot (Fig. 1 A).

In total, we found 5,792 variants in 537 nominated target genes (Table S1, A and B). Across all three wild-derived mice in this study, we found genotyped variants in 401 of the total 537 nominated target genes. While a large portion of these variant calls was identical across CAST, PWK, and WSB mice (2,601 out of 5,792), there were a number of strain-specific variants. For example, within inositol polyphosphate-5-phosphatase D (*Inpp5d*;chr1:87620312–87720507), there were five genotyped variants (Fig. 1 B). One SNP was shared between all three wild-derived mice, three SNPs were shared only by CAST and PWK, and one SNP was heterozygous in CAST and PWK but homozygous in WSB (Table S1 C). This demonstrates the genetic heterogeneity of these mouse models within key genes studied in AD.

Cytochrome P450 3A43 (*Cyp3a43*; chr5: 137890932–146113285) contained the most genotyped variants with 563, only 163 of which were shared among all wild-derived mice. 136 of the 537 AMP-AD target genes did not contain any genotyped variants. More information about all variants in these wild-derived mice is available on the Mouse Genome Database (http://www.informatics.jax.org). While our description is limited to those variants genotyped by our selected Illumina Infinium platform, these data suggest that the genetic heterogeneity of the wild-derived mouse genetic backgrounds could modulate many genes of interest for the study of AD and related dementias.

### Pilot study to determine sample size for viral approach

To preserve the effect of genetic background, we selected to model tauopathy with a viral approach. Expressing mutant tau without the need to backcross allows us to test the effect of a “pure genetic background,” without the need to regenotype each experimental mouse for all variants of interest. We used an AAV-mediated gene expression model, as described before (Carlomagno et al., 2019; Cook et al., 2015; Kim et al., 2008).

**Figure S1.**
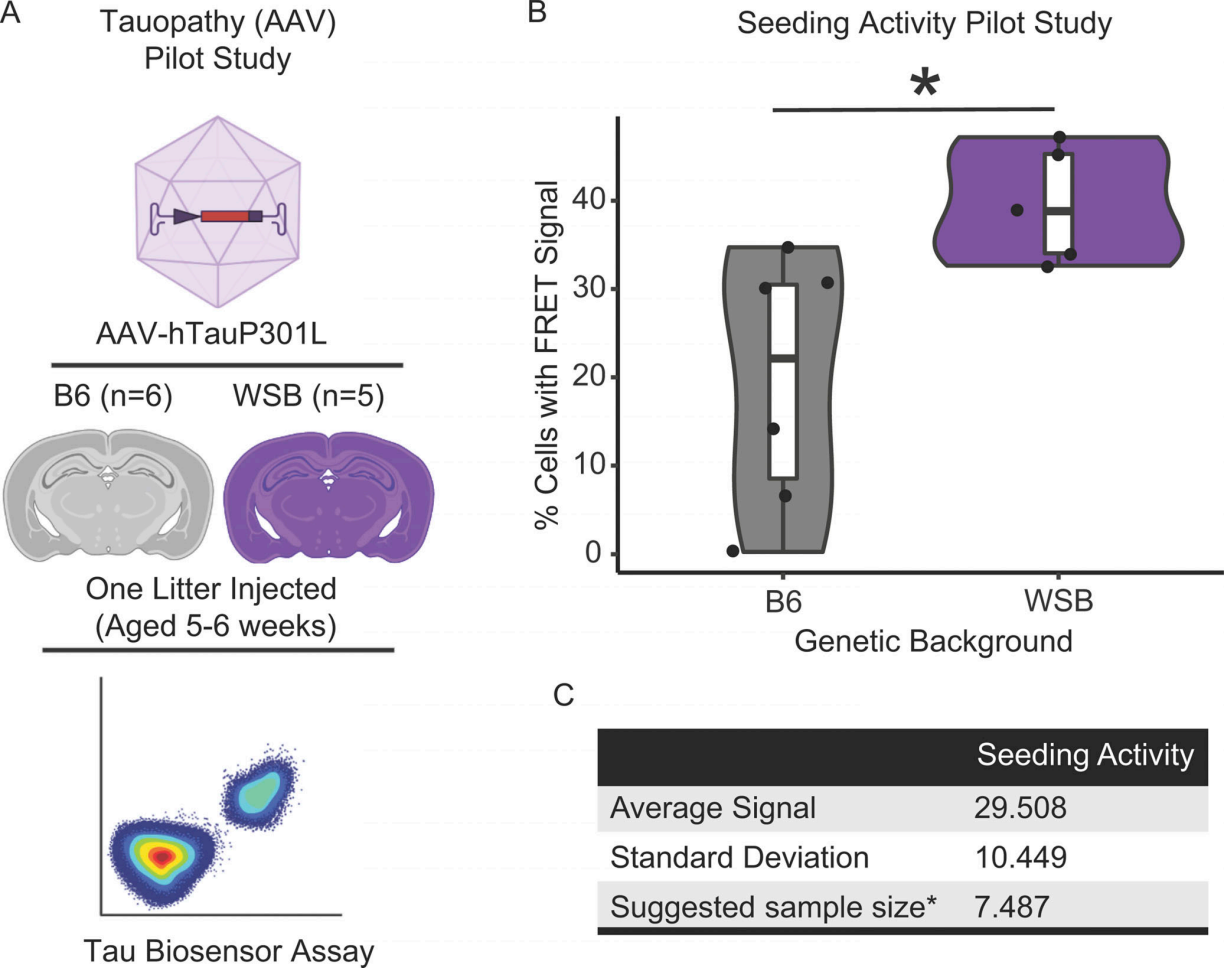
**Pilot study to calculate the sample size for the tau seeding assay. (A)** Design of a pilot study to determine the sample size. One litter of B6 and WSB mice was injected with AAV-hTauP301L and aged 6 wk. TBS-soluble protein lysate from the cortex of each pup was transfected into tau biosensor cells. 24 h after transfection, cells were trypsanized and FRET+ signal was measured via FACS as a proxy for tau seeding activity. **(B)** Percent cells with FRET signal were significantly increased in WSB versus B6 in our pilot study (*P < 0.05; n_B6_ = 6, n_WSB_ = 5; Welch’s *t* test P = 0.0172). **(C)** The sample size was calculated based on the standard deviation observed in our pilot study with the criteria of power = 0.8, alpha = 0.05, group = 4, effect size = 20% of average signal, variation = standard deviation. *Sample size determined in the R Stats Package using power.anova.stats(). This analysis indicates that at least eight mice per group are needed to properly power the main study.

To ensure that we would be statistically powered to test the effect of genetic background on tau seeding, we designed a pilot experiment. One litter of B6 and WSB mice was injected with AAV-hTauP301L. At 6 wk of age, we then evaluated the effects of genetic background on tau seeding activity using the tau seeding assay biosensor assay. Using an effect size of 20% for the FRET+ (fluorescence resonance energy transfer) signal, a power of 0.8, a group number of 4, and P < 0.05, we aimed for a final sample size of at least eight AAV-hTauP301L–injected mice per genetic background (Fig. S1, A–C).

### Tau seeding activity is significantly increased on CAST and PWK genetic background independent of tau expression level

**Figure 2.**
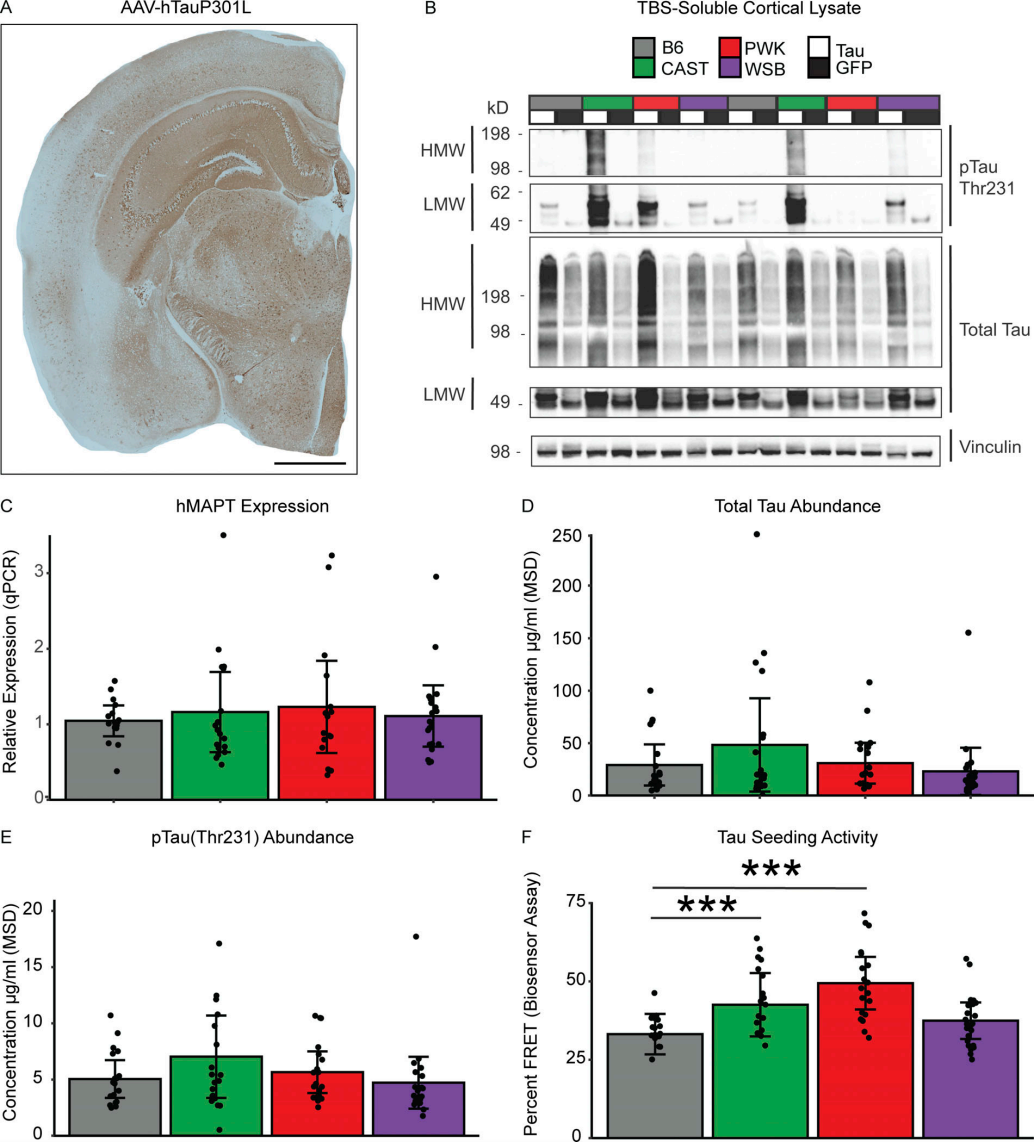
**Tau seeding activity is modulated by genetic background independent of tau expression level. (A)** Representative image shows a widespread expression of human tau (HT7+ stain) in AAV-hTauP301L–injected mice (scale bar 1 mm). **(B)** Representative Western blot shows high molecular weight (HMW) and low molecular weight (LMW) bands of total tau (TOMA+) and pTau Thr231 in the cortex of AAV-hTauP301L–injected mice compared to AAV-eGFP–injected controls (*n* = 2/condition). Quantification of Tau levels across genetic backgrounds was performed using two independent experiments: viral expression of human tau using qPCR and abundance of total tau via Meso Scale Diagnostics Total Tau Kit. **(C)** Human tau expression was measured using qPCR. Relative hMAPT expression was calculated relative to GAPDH and showed no effect of genetic background via one-way ANOVA (*n* = 16–20 per group, technical replicates = 2, F_3,66_ = 0.234, P = 0.87). **(D and E)** Total tau and pTau Thr231 levels were measured via Meso Scale Diagnostics (MSD; K15121D). There was no effect of genetic background on either total tau via one-way ANOVA (*n* = 19–21 per group, F_3,75_ = 1.439, P = 0.238) or pTau231 (*n*= 19–21 per group, F_3,75_ = 1.665, P = 0.189). **(F)** To measure tau seeding activity, an in vitro biosensor assay was performed. HEK-293T cells containing CFP- or YFP-conjugated tau are transfected with brain lysate from hTauP301L-injected mice for 24 h. Biosensor cells are then collected and FRET+ signal is measured via FACS as a proxy for tau seeding activity. Tau seeding activity was significantly affected by genetic backgrounds via one-way ANOVA (*n* = 19–24 per group, technical replicates =2, F_3,78_ = 9.237, P = 2.67 × 10^−5^). Tukey honest significant difference post-hoc test revealed elevated tau seeding activity in CAST and PWK relative to B6 (***P < 0.001; B6-CAST *p_adj* = 0.046, B6-PWK *p_adj* = 1.12 × 10^−4^). Source data are available for this figure: SourceData F2.

**Figure S2.**
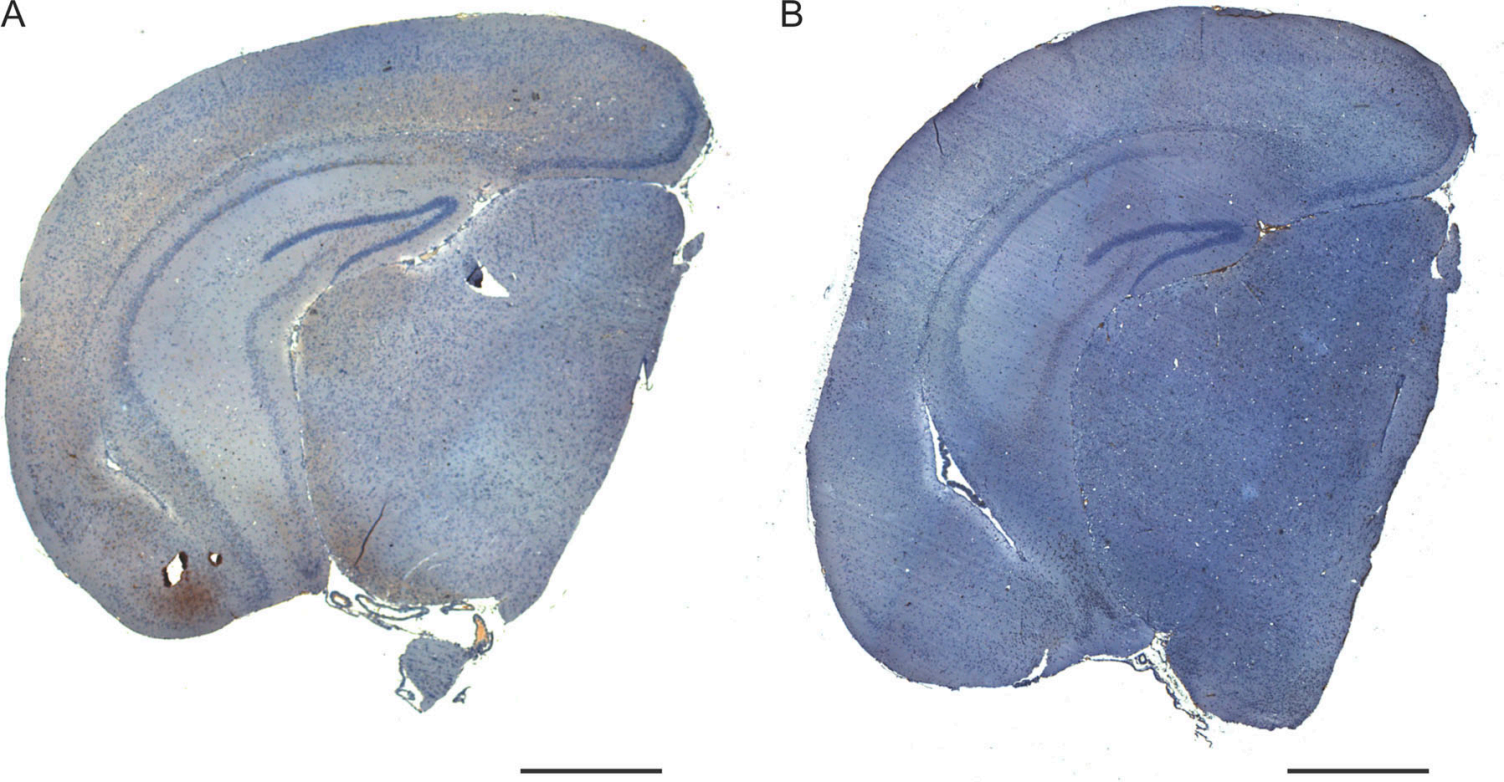
**Human tau specific expression in AAV-hTauP301L–injected mice relative to AAV-eGFP–injected controls. (A and B)** Representative images show expression of human tau (HT7+ stain) in AAV-hTauP301L–injected mice (A; scale bar 1 mm) compared with AAV-eGFP–injected control (B; scale bar 1 mm).

To determine tau expression in inbred mice strains, mice injected with either AAV-hTauP301L or AAV-eGFP were harvested at 6 mo of age, and the human tau specific expression was assessed first via histology using HT7 human tau antibody (Fig. 2 A). Consistent tau expression was observed in the cortex of AAV-hTauP301L–injected mice (Fig. S2 A) but not in AAV-eGFP–injected mice (Fig. S2 B). Next, we confirmed the tau pathology in AAV-hTauP301L–injected mice with an independent method via immunoblotting (Fig. 2 B). Representative samples demonstrate the presence of high molecular weight and low molecular weight species of phospho-tau Thr231 and total tau (DAKO).

To determine consistency in viral expression of the hTauP301L, we measured human *MAPT* expression from the cortex of B6, CAST, PWK, and WSB mice. We found no significant effect of genetic background on the human *MAPT* mRNA levels (Fig. 2 C; F_3,66_ = 0.234, P = 0.87, *n* = 70). To quantify the amount of tau protein, we measured total tau and pTau231 concentration in TBS-soluble lysate from the cortex of B6, CAST, PWK, and WSB mice. We found no effect of genetic background on levels of total tau (Fig. 2 D; F_3,75_ = 1.439, P = 0.238, *n* = 19–21 per group) or pTau231 (Fig. 2 E; F_3,75_ = 1.665, p 0.189, *n* = 19–21 per group). These data suggest that the genetic variations across different mouse genetic backgrounds do not influence our ability to express tau using AAV-hTauP301L injection.

To investigate whether the pathogenesis of tau aggregates differs across genetic backgrounds, we measured the proteopathic tau seeding activity using an in vitro biosensor cell assay. A cell line expressing the repeat domain (RD) of tau conjugated to either a cyan fluorescent protein (CFP) or yellow fluorescent protein (YFP) was transfected with brain lysate from AAV-hTauP301L mice of each genetic background. FRET signal occurs when tau seeds form due to the proximity of CFP and YFP molecules. FRET+ signal is then measured by fluorescence-activated cell sorting (FACS) as a proxy for tau seeding activity. Sample size was determined based on our pilot experiment to specifically power the study for this assay (Fig. S1, A–C). Interestingly, genetic background significantly affected tau seeding activity when we used cortical tissue lysates as seeding agents. Percent FRET+ events measured by flow cytometry were modulated by genetic background (Fig. 2 F; F_3,78_ = 9.237, P = 2.67 × 10^−5^_,_ *n* = 19–23 per group). CAST and PWK genetic background mice had a significant increase in tau seeding activity compared to B6 controls (P < 0.001). However, there was no significant difference between B6 and WSB AAV-hTauP301L–injected mice (P > 0.05). Taken together, our data suggest that the genetic heterogeneity across wild-derived mice exacerbates the prion-like action of tau in the brain cortex.

### Signature A: Core tau-responsive signature across genetic backgrounds

**Figure 3.**
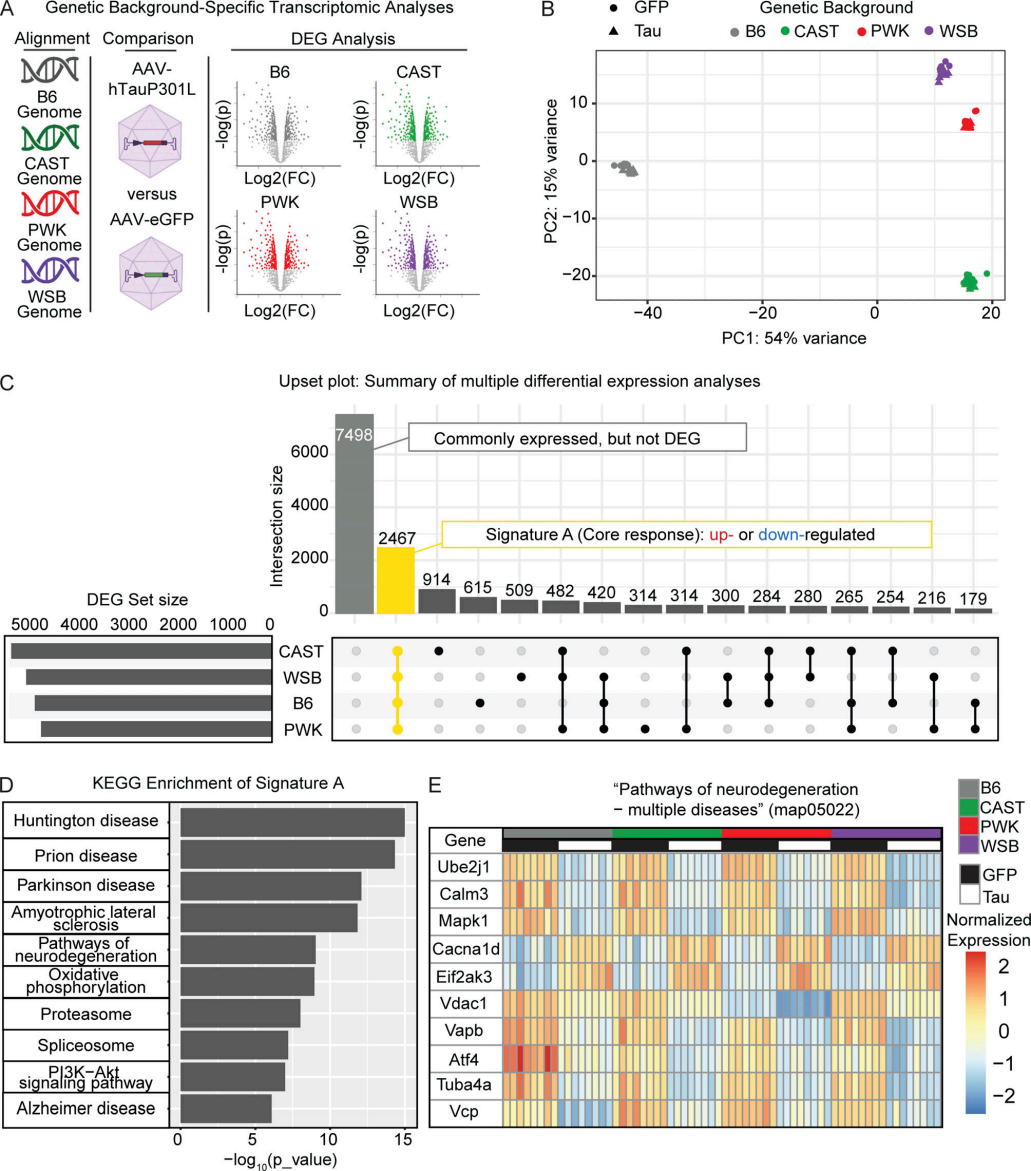
**Signature A: Core tau-responsive signature across genetic backgrounds. (A)** Experimental design to express tau in B6 and three wild-derived mouse strains. AAV-eGFP or AAV-hTauP301L was injected into mice of each genetic background. At 6 mo, brain tissue was collected and analyzed via mRNA-seq. Reads were aligned to each strain’s respective genomes. Differential gene expression revealed upregulated (fold change [FC] > 1.5, Benjamini Hochberg P_adj < 0.05) and down-regulated (FC < −1.5, Benjamini Hochberg P_adj < 0.05) in hTauP301L-injected mice compared to GFP-injected controls (*n* = 32/AAV injection group). Volcano plots for illustration purpose only, please see supplemental information Fig. S3, A–D, and Table S2, A–D. **(B)** PCA shows the genetic background drives variation in the transcriptome (*n* = 8/background/AAV injection group). **(C)** Upset plot to summarize multiple differential expression analyses: Differential expression (hTauP301L vs. eGFP) was performed for each strain (see Fig. S1 and Table S2, A–D). Signature A (highlighted in yellow) was identified as the intersection of DEGs shared across genetic backgrounds. Other intersections are provided as a resource (Table S2 E). **(D)** KEGG enrichment of Signature A is significantly enriched for neurodegeneration-related terms and Pathways of neurodegeneration (map05022; 168 DEGs in Signature A out of 471 genes in map05022). See the supplemental information for a summary of all enrichment analyses (Table S2 J). **(E)** Heatmap of the top 10 DEGs in Pathways of neurodegeneration—multiple diseases (map05022) shows the conserved response to AAV-hTauP301L injection in Signature A.

To understand the effect that genetic background has on modeling the expression of human tau, we performed bulk RNA-seq on the cortex of 6-mo-old mice injected with either AAV-hTauP301L or AAV-eGFP (Fig. 3 A; *n* = 8/background, respectively). Principal component analysis (PCA) demonstrates that the largest contribution to the variation in the transcriptome is genetic background (Fig. 3 B). These data suggest that genetic variation across genetic backgrounds is the greatest driver of gene expression. To define differentially expressed genes (DEGs) between AAV-hTauP301L– and AAV-eGFP–injected mice, we used adjusted P < 0.05 and a 1.5-fold cutoff for up- or downregulated genes.

**Figure S3.**
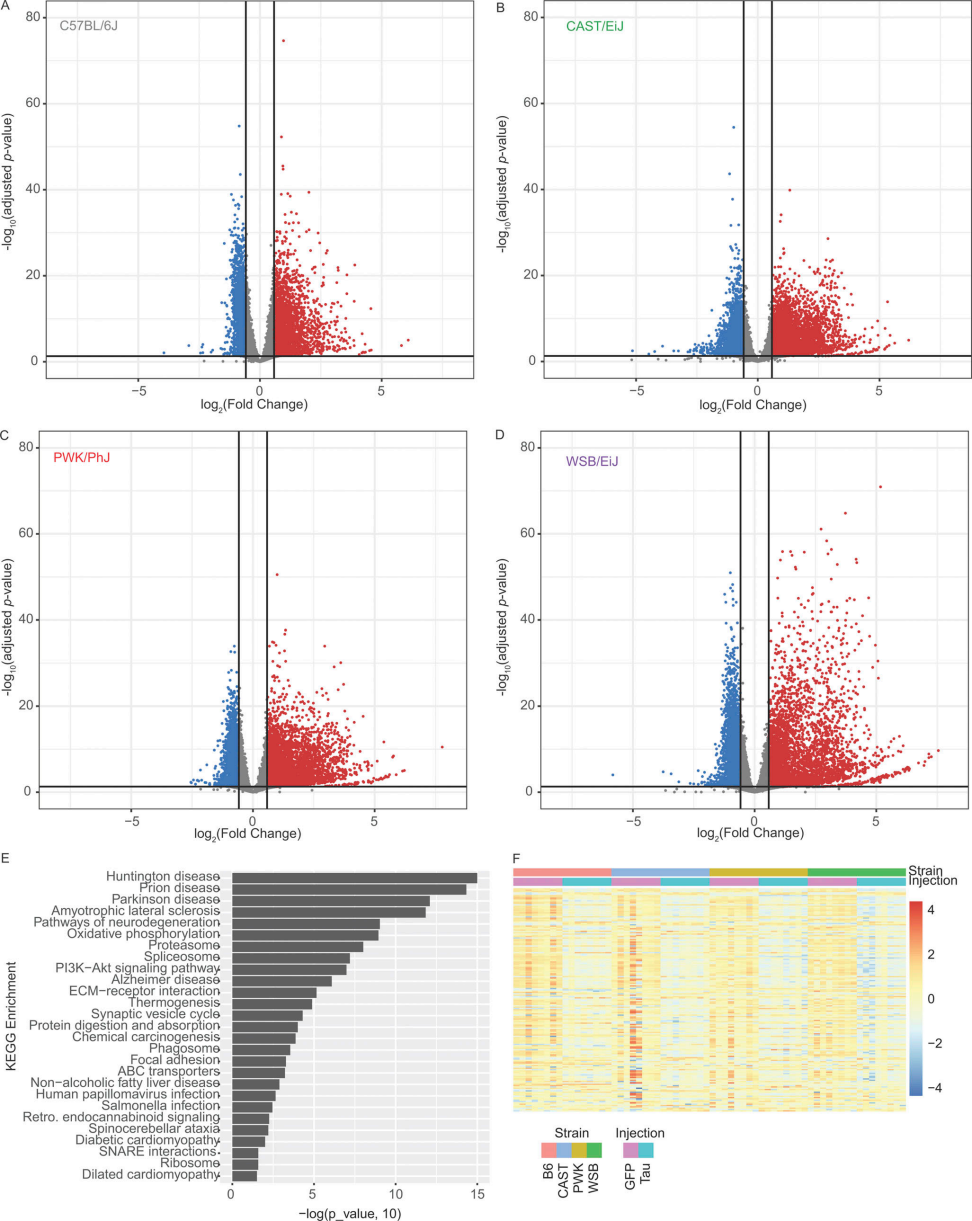
**Transcriptomic analyses for discovery of core tau response (Signature A).** Volcano plot demonstrates the log_2_ fold change (x axis) and statistical significance (Benjamini-Hochberg adjust P value, y axis). **(A–D)** (A) B6, (B) CAST, (C) PWK, and (D) WSB mice were analyzed separately to compare genes upregulated (red, fold change [FC] > 1.5, P_adj < 0.05) and downregulated (blue, FC < 1.5, P_adj < 0.05) in tau-injected mice compared with GFP-injected controls (*n* = 32/injection group). **(E)** KEGG enrichment of Signature A defined in Fig. 2 C. **(F)** Heatmap of all Signature A genes in KEGG map05022.

Comparisons were made between AAV-hTauP301L– and AAV-eGFP–injected mice for each genetic background independently (Fig. S3, A–D; and Table S1, A–D). There are a number of genes that are specific to each genetic background (Lilue et al., 2018). Our resource only includes genes that were identified with at least 10 total read counts across all samples of a given genetic background. We identified a total of 4,784 DEGs in B6; 5,260 DEGs in CAST; 4,657 DEGs in PWK, and 4,958 DEGs in WSB (DEG set size, Fig. 3 C). Of these DEGs, 2,467 genes were commonly expressed across all genetic backgrounds and were identified as DEGs in all genetic backgrounds (yellow highlighted intersection, Fig. 3 C). The upset plot also shows genes that were commonly expressed but not DEGs and DEGs shared between different combinations of genetic backgrounds (Fig. 3 C). Gene sets unique to each background (i.e., CAST-only DEGs, *n* = 914) are available in Table S2 E. These data suggest a large part (2,467 genes) of what we call the “Signature A: core tau signature” is resistant to the variation between genetic backgrounds.

To understand which genes are part of Signature A, we performed enrichment analyses. Kyoto Encyclopedia of Genes and Genomes (KEGG) enrichment analysis of this gene set (2,467 genes) was significantly enriched for several neurodegenerative terms (Fig. 3 D, Fig. S3 E, and Table S2 J). As an example, the KEGG term “Pathways of neurodegeneration—multiple diseases” demonstrates a pathway that is conserved across all genetic backgrounds in this study (Fig. 3 E). A total of 168 genes out of the 471 genes in the pathway (Pathways of neurodegeneration—multiple diseases KEGG map05022) follow this genetic background–independent effect (Fig. S3 F). For example, *Ube2j1*, *Calm3*, and *Mapk1* genes were lowly expressed similarly in B6 and all three wild-derived mice injected with AAV-hTauP301L compared with the AAV-eGFP–injected control group (Fig. 3 E). These data demonstrate that the core tau signature includes many targets that are already implicated in neurodegenerative diseases.

### Signature B: Tau-responsive signature unique to wild-derived genetic backgrounds

While the presence of DEGs is informative when comparing genetic backgrounds, we were also interested to discover novel targets for tauopathy that may be present only in wild-derived mice. To do this, we performed DEG analysis using the genetic background as a covariate with injection type (∼Injection+GeneticBackground+Injection:GeneticBackground). This approach differs from the differential expression analysis to identify Signature A as it identifies DEGs that are not shared across the genetic background in response to tau. A total number of 79 DEGs were identified in CAST (Effect: tau.CAST; Table S1 F), 51 DEGs in PWK (Effect: tau.PWK; Table S1 G), and 53 DEGs in WSB (Effect: tau.WSB; Table S1 H). These data suggest that there exist some novel responses to tau present only in wild-derived mice.

**Figure 4.**
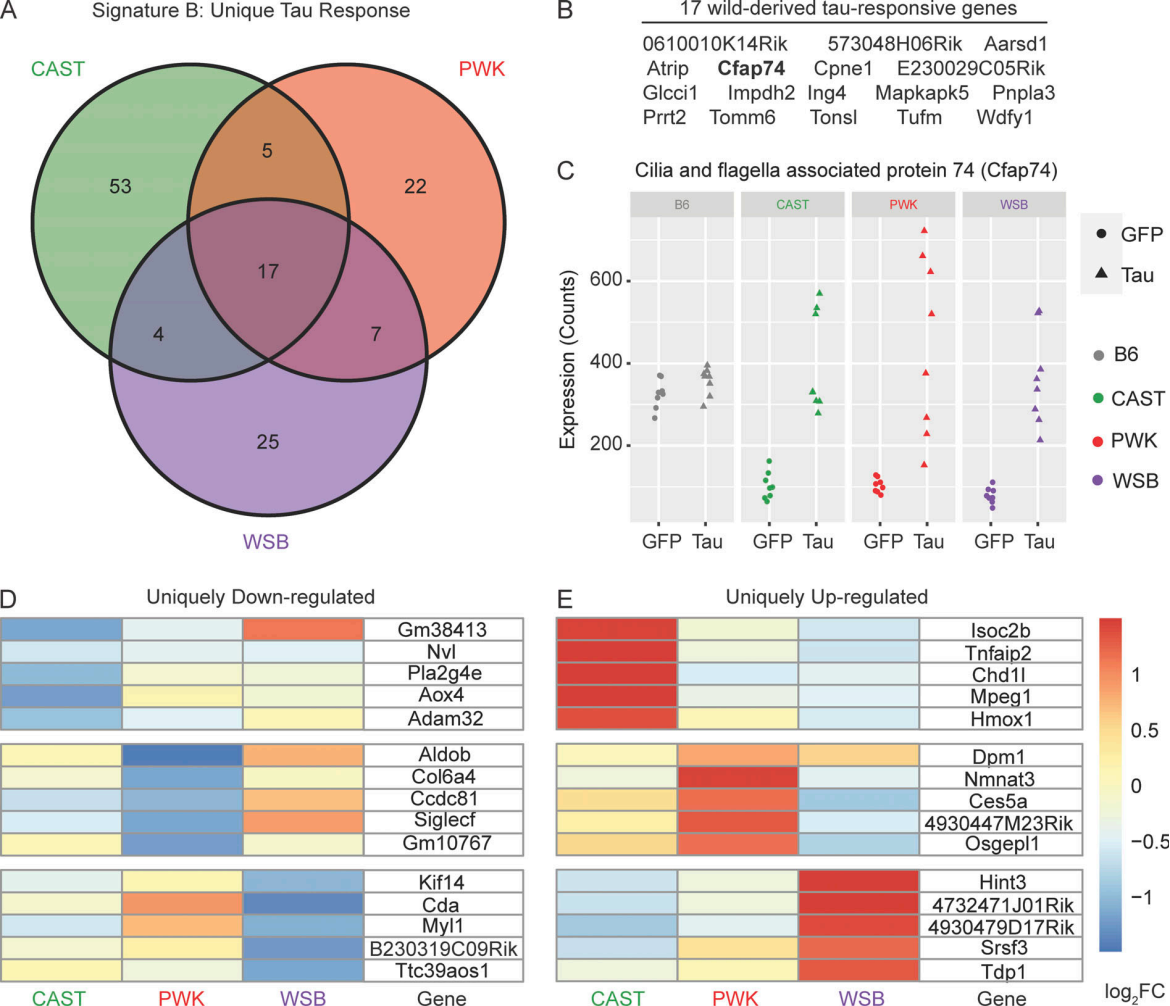
**Signature B: Tau-responsive signatures unique to wild-derived genetic backgrounds.** DEGs specific to wild-derived background (∼Injection+GeneticBackground+Injection:GeneticBackground; Benjamini Hochberg adjusted P value <0.05, fold change > 1.5) were calculated for hTauP301L-injected mice relative to eGFP-injected controls. **(A)** 133 in total DEGs were identified in one or more wild-derived backgrounds. **(B)** 17/133 DEGs in Signature B were shared by all three wild-derived backgrounds. **(C)** Cilia- and flagella-associated protein 74 (*Cfap74*) and 16 other wild-derived DEGs are not differentially expressed in B6 mice. There are 53 CAST-specific DEGs, 22 PWK-specific DEGs, and 25 WSB-specific DEGs. **(D and E)** The (D) top five downregulated and (E) top five upregulated in each background are shown in a heatmap colored by log_2_FoldChange between hTauP301L-injected and eGFP-injected mice. See supplemental files for all background-specific DEGs (Table S2, F–H).

By deciding to calculate DEGs with a genetic background as a covariate, we were able to identify tau response genes that are specific to the wild-derived strains. A Venn diagram of the DEGs shows how many genes are shared across these wild-derived strains (Fig. 4 A). There were 17 genes shared by CAST, PWK, and WSB (Fig. 4 B) that were not differentially expressed in B6 mice. As an example, cilia- and flagella-associated protein 74 was upregulated in wild-derived mice injected with AAV-hTauP301L compared with AAV-GFP–injected mice of the same genetic background (Fig. 4 C). As more risk genes are characterized in the study of tauopathy, it is critical that these genes can be modeled on backgrounds other than B6.

Uniquely downregulated (Fig. 4 D) or upregulated (Fig. 4 E) DEGs in just one wild-derived strain is a rare phenomenon, especially in comparison to Signature A, which is comprised of 2,467 DEGs. While there has been no precedent for private DEGs that have a large effect on modeling tauopathy, our data show some divergent responses to tau. Although the number of wild-derived specific DEGs in Signature B is not large enough to reach significance in enrichment analyses, taken together, these 133 genes in Signature B are enriched for several pathways that have not been studied well in the context of tauopathy (Table S2 K). The significantly enriched KEGG pathways, such as asthma (KEGG:05310) and *Staphylococcus aureus* infection (KEGG:05150), are not obviously associated with the study of tauopathy.

### Network analysis identified darkorange module as a putative mediator of tau seeding

**Figure S4.**
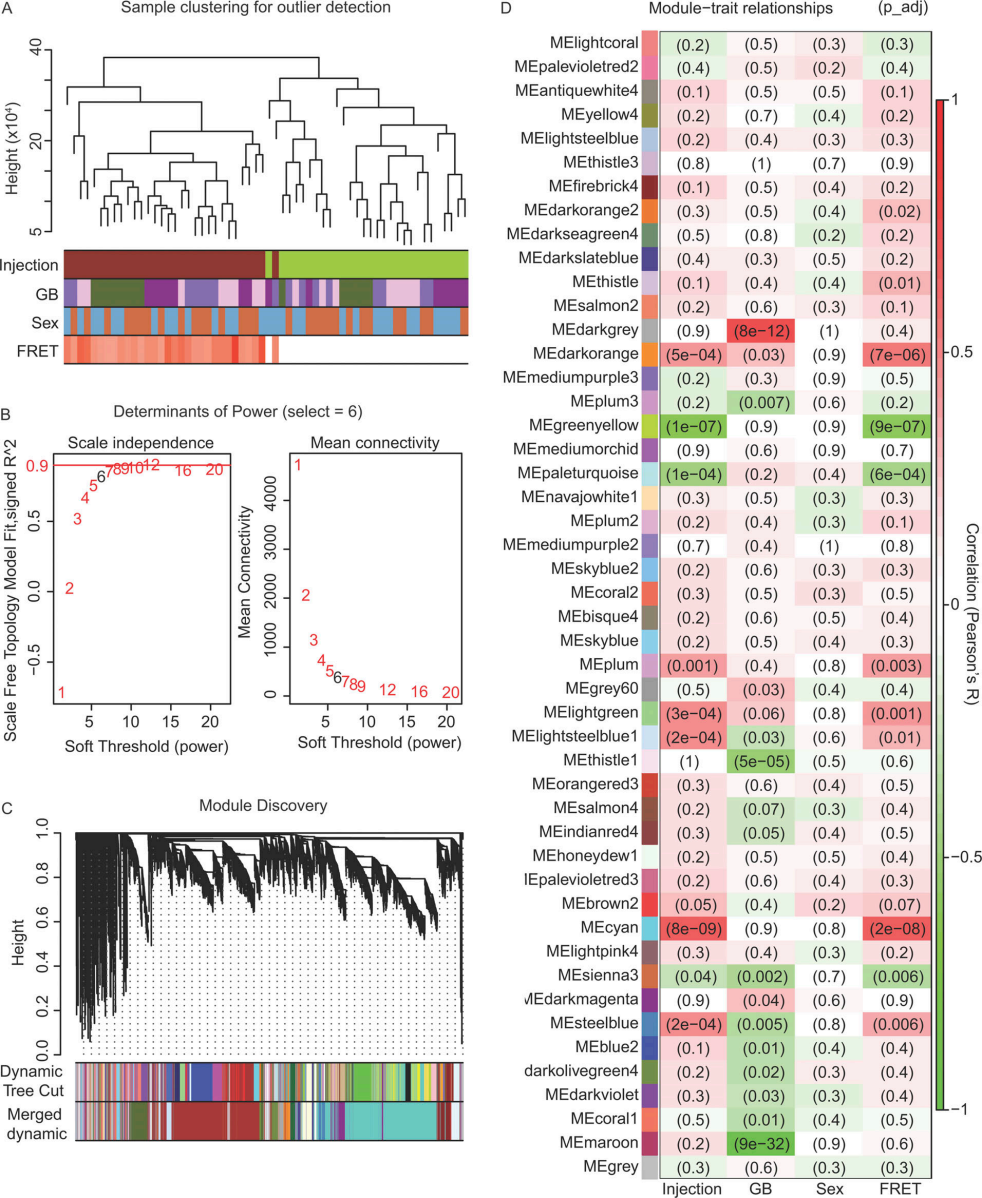
**Summary of WGCNA.** Gene module detection was performed using WGCNA from hTauP301L-injected and eGFP-injected mice. **(A)** Sample dendrogram and trait heatmap reveal outliers by calculating unbiased sample similarity. Trait heatmap shows samples are segregated out mainly by injection type and seeding activity score (FRET) instead of sex or genetic background (GB). **(B)** Scale independence and mean connectivity calculated by the WGCNA package. Although no thresholds reached the recommended 0.9 threshold for scale free topology, a threshold of 6 was selected based on recommendations of the package’s authors for unsigned network detection in an experiment with at least 40 samples. **(C)** Module discovery was performed by clustering genes based on topological overlap matrix dissimilarity (y axis: height). Similar clusters were merged using a dissimilarity threshold of 0.25 (merged dynamic). 60 remaining clusters were assigned arbitrary names using R’s color palette. **(D)** To prioritize modules of interest, quantified traits were correlated to each module’s eigengene expression. Benjamini-Hochberg adjusted P values were reported in each cell of the heatmap (colored by Pearson’s R). Injection is defined as a binary trait (1: tau, 0: GFP). Genetic background is defined as a binary trait (1: wild-derived, 0: B6). Sex is defined as a binary trait (1: female, 0: male). FRET is defined as a measurement of % cells with FRET+ signal from Fig. 4 C.

Given the significant difference in tau seeding activity across genetic backgrounds, we aimed to identify gene expression signature–associated tau seeding. We performed weighted gene co-expression network analysis (WGCNA) to investigate which genes may be correlated with this difference in seeding activity using our FRET data as a trait for “module-trait” relationship analyses. We identified 60 modules of co-expressed genes (Table S1 I and Fig S4, A–C) and tested their correlation to traits including whether mice were injected with AAV-eGFP or AAV-hTauP301L (Injection), whether mice were B6 or one of the wild-derived genetic backgrounds (GB), biological sex (Sex), and seeding activity (FRET). Of these 60 modules, 21 were significantly associated with either seeding activity or wild-derived genetic background (Fig. S4 D, P < 0.05). Importantly, none of these 21 modules demonstrated a significant effect of sex (Fig. S4 D).

**Figure 5.**
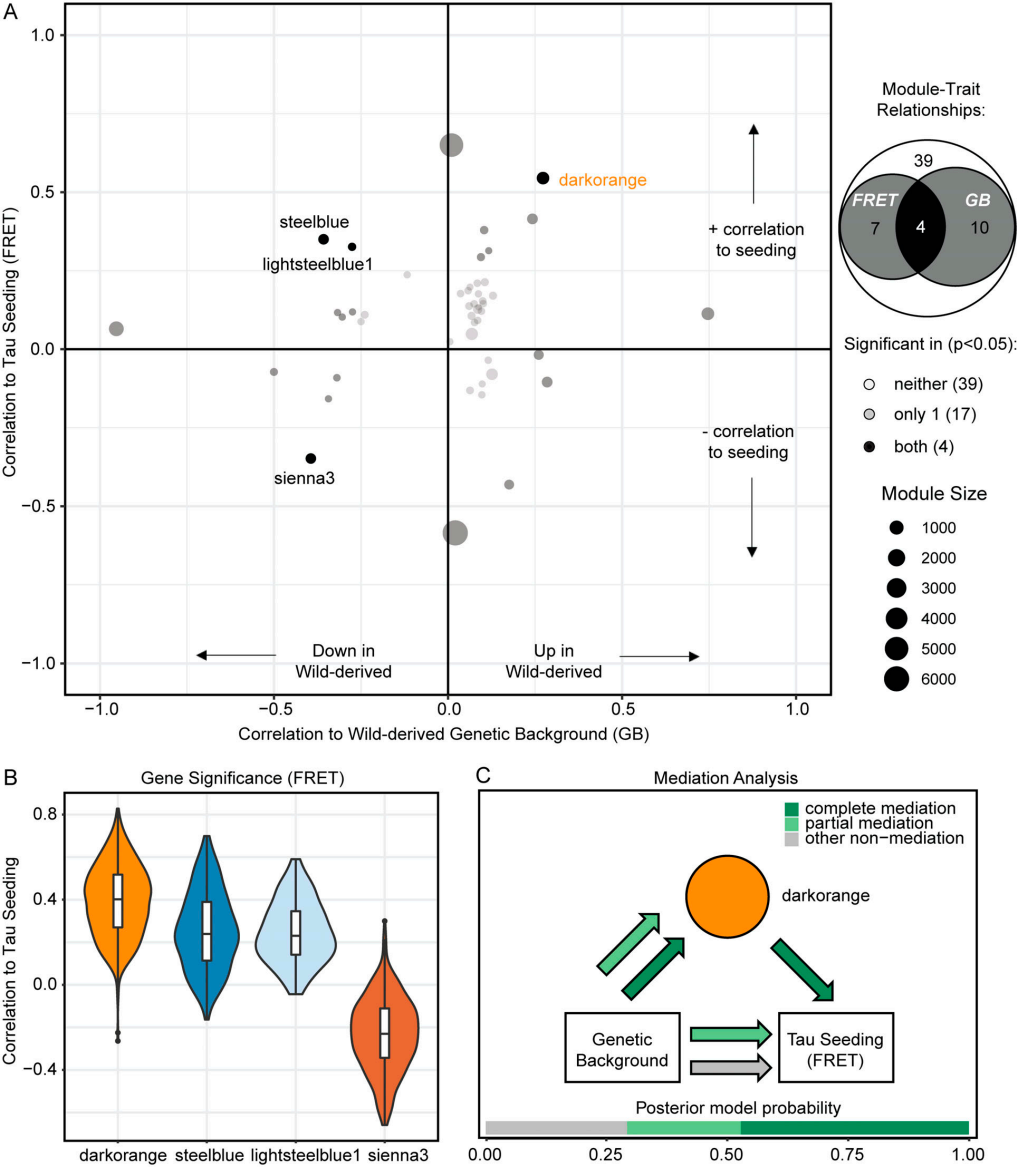
**Network analysis identified darkorange module as putative mediator of tau seeding.** Gene module detection was performed using WGCNA from hTauP301L-injected and eGFP-injected mice. See supplemental information for WGCNA parameters (*n* = 60 samples after outlier detection). A full list of all 60 modules and their module–trait correlation can be found in Fig. S3. **(A)** Two-dimensional module-trait detection reveals four modules statistically significant for both the correlation to tau seeding (FRET) and the correlation to the wild-derived genetic background. Pearson correlations were performed in the WGCNA package per default parameters and considered significant with a Benjamini Hochberg adjusted P value <0.05. Points on the scatter plot are sized according to the number of genes in each module and the opacity set by whether the module is statistically significant for both FRET and GB, either trait, or neither trait. **(B)** Gene significance (GS) is calculated for every gene in WGCNA analysis. Plotting GS by the module of interest reveals that the darkorange module contains the most genes positively correlated to tau seeding. **(C)** Representation of mediation analysis was performed on a matrix of eigengenes for each module. Two steps of mediation analysis identified that darkorange is a significant mediator of the relationship between genetic background and tau seeding (joint significance calculated via HIMA, P = 0.0349). Furthermore, Bayesian model selection suggests that the relationship between the darkorange module and tau seeding is most probabilistically via complete mediation (dark green arrows).

Each of these 21 modules with a significant Module:Trait_FRET_ relationship may contain genes that explain the increase in seeding activity seen in CAST and PWK mice. To prioritize modules significant for both seeding activity (FRET) and wild-derived genetic background (GB), we focused on those that were significantly associated via Pearson correlation (Fig. 5 A, R: positively correlated 0–1, negatively correlated −1 to 0). Using this approach, we were able to identify four modules that were significantly correlated to both traits of interest: darkorange, steelblue, lightsteelblue1, and sienna3 (Fig. 5 A). The darkorange module is the only one of these four modules of interest to be positively correlated to FRET measurement (Fig. 5 A, R^2^ = 0.54, P = 7 × 10^−6^) and showed a positive correlation to wild-derived background (R^2^ = 0.27, P = 0.03). Gene significance calculation of each gene in the four modules of interest reveals the genes within the darkorange module were most positively correlated to tau seeding (Fig. 5 B).

To investigate whether the genes in the darkorange module were putative mediators of the relationship between genetic background and tau seeding, two independent pipelines were used to perform mediation analyses. First, high-dimensional mediation analysis (HIMA; Zhang et al., 2016) was performed to evaluate which of the WGCNA modules were putative mediators. The darkorange module was identified as a significantly associated mediator (α = 0.0833, β = 35.7258, P = 0.0349). Additionally, we sought to describe the relationship between genetic background (the independent variable), seeding activity (the dependent variable), and the darkorange module (the plausible mediator) using a Bayesian module selection approach (Crouse et al., 2022). Using the default effect size priors and the “complete” model options of the bmediatR package, we calculated the posterior model probability for complete mediation, partial mediation, and other non-mediation (Fig. 5 C). Of these three possible relationships between genetic background, seeding activity, and the darkorange as a mediator, we found that the darkorange module is most likely to act through complete mediation. For this reason, we focused on the darkorange module to describe the effect of genetic background on tau seeding activity “Signature C.”

### Signature C: Darkorange module implicates microglia in response to tau seeds

**Figure 6.**
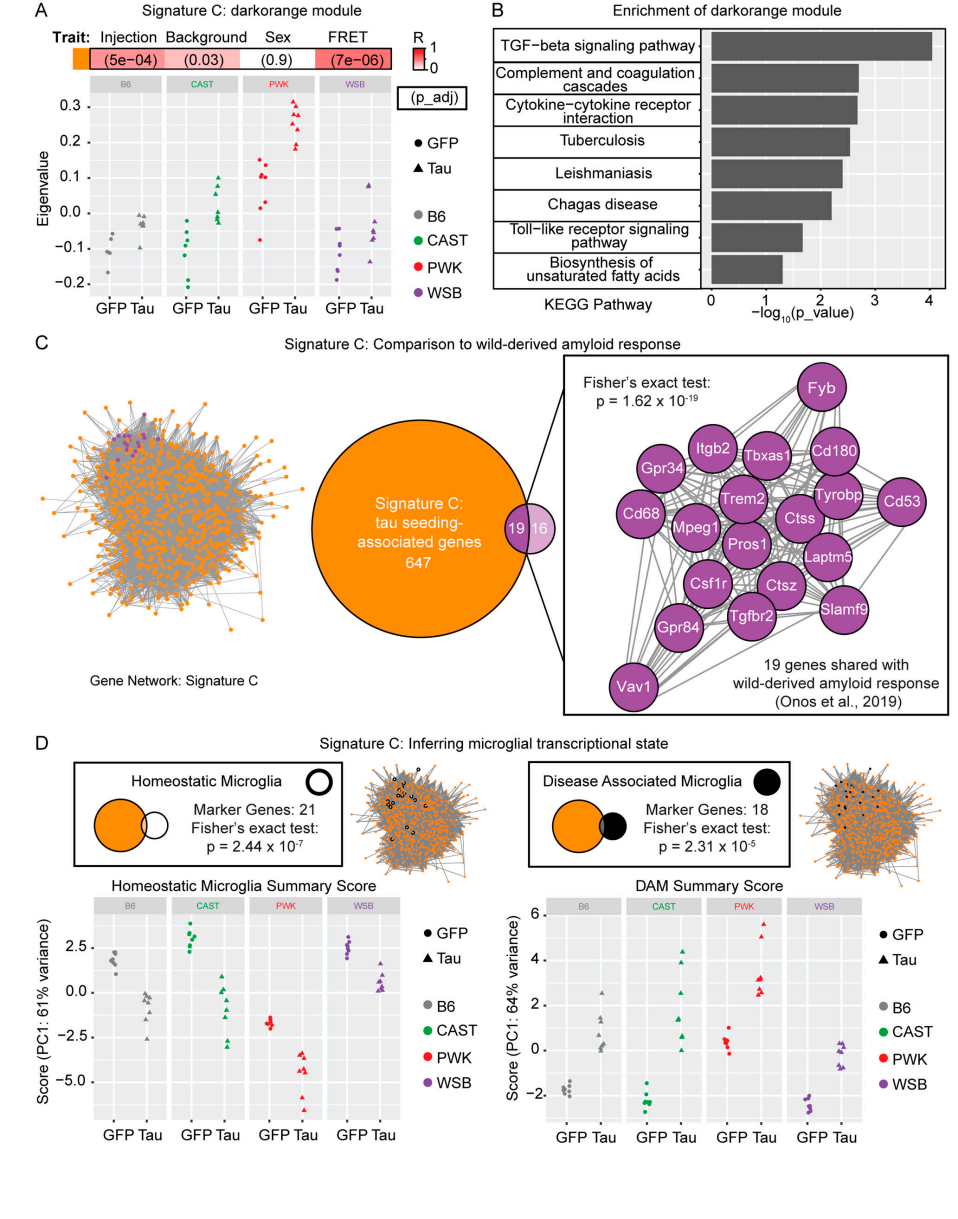
**Signature C: Darkorange module implicates microglia in response to tau seeds. (A)** Darkorange module was renamed Signature C and the eigengene value of the darkorange module for each mouse was calculated as part of the WGCNA pipeline. Differences observed in eigengene expression follow a similar pattern to tau seeding activity, with the largest increases observed in CAST and PWK mice injected with AAV-hTauP301L. **(B)** KEGG enrichment of Signature C reveals microglia-related terms calculated in gprofiler2 via Fisher’s one-tailed test (all terms P < 0.05). **(C)** Signature C gene network is represented as nodes with edge distance representing topological overlap matrix score from WGCNA. Neurodegenerative hub-genes (purple) were detected by comparing Signature C (darkorange) and wild-derived amyloid response (Fisher’s exact test: P = 1.62 × 10^−19^; Onos et al., 2019). **(D)** Microglial transcriptional state was inferred using previously published markers for homeostatic (Li et al., 2019) and disease-associated microglia (Keren-Shaul et al., 2017). The homeostatic microglial signature was significantly enriched (Fisher’s exact test: P = 2.44 × 10^−7^) and showed the largest decrease in PWK injected with AAV-hTauP301L. The disease-associated microglial signature was significantly enriched (Fisher’s exact test: P = 2.31 × 10^−5^) and shows the largest increase in PWK injected with AAV-hTauP301L.

The eigenvalue of the expression of genes in the darkorange module is increased in AAV-hTauP301L–injected mice relative to AAV-eGFP–injected controls (Fig. 6 A). Furthermore, the eigenvalue of genes in this module follows a similar pattern to the tau seeding findings, with CAST and PWK mice showing increased expression relative to B6 and WSB. We further characterize this tau seeding-associated signature using enrichment analysis. Multiple immune responses (Fig. 6 B) were identified using KEGG. These data suggest that the immune system may be implicated in the increase in seed-competent tau observed in CAST and PWK mice. Additional enrichment analyses of Signature A, Signature B, and Signature C can be found in Table S2, J–L.

The darkorange module contains a total of 666 genes that we found to be associated with the effect of genetic background on tau seeding activity (Fig. 6 C, highlighted in orange). A similar effect of the genetic background was found via WGCNA when modeling Aβ (Onos et al., 2019). In comparison to their 35-gene module, we found that 19 of their genes were significantly enriched also in our darkorange module (Fig. 6 C, highlighted in purple; Fisher exact test, P = 1.62 × 10^−19^). Interestingly, key microglial genes (*Trem2*, *Tyrobp*, *Tgfbr2*) and the complement cascade genes (*C1qa*, *C1qb*, *Pros1*) were affected by genetic background in both amyloid and tau studies (Fig. 5 C). This finding suggests that both pathways are sensitive to genetic context when modeling both hallmarks of AD.

Since the darkorange module was enriched for innate immune response and overlapped with the previously published wild-derived responsive genes implicated in microglial function, we investigated whether markers of homeostatic or disease-associated microglia were present in this module. We selected marker genes previously identified via single-cell RNA-seq for homeostatic microglia (Li et al., 2019) and disease-associated microglia (Keren-Shaul et al., 2017). We found 21 homeostatic marker genes (Fisher’s exact test: P = 2.33 × 10^−7^) and 18 disease-associated marker genes (Fisher’s exact test: P = 2.31 × 10^−5^) within the darkorange module. The expression score of each signature via PCA of raw reads demonstrated similar shifts in PWK and CAST, which were concordant with the changes observed in tau seeding activity (Fig. 6 D). Although we are inferring the microglial transcriptional state from bulk transcriptomic data, there is a clear shift in homeostatic and disease-associated marker genes across the genetic background when hTauP301L is expressed.

### Signature C: Darkorange module genes in shared inflammatory response to tau

To identify which darkorange genes may regulate the immune response to tau, we performed targeted gene expression analysis using the mouse neuroinflammation panel (nCounter Nanostring; *n* = 3/genotype/injection, females). This panel consists of 757 genes covering multiple pathways of neuroimmune response, neuropathology, and metabolism. We found that ∼8% (56/666) of the genes in the darkorange module were selected as part of this curated list (Table S2 R). We hypothesized that this subset of genes already implicated in neuroinflammation could provide valuable insights into the inflammatory response to tau in wild-derived mice.

**Figure 7.**
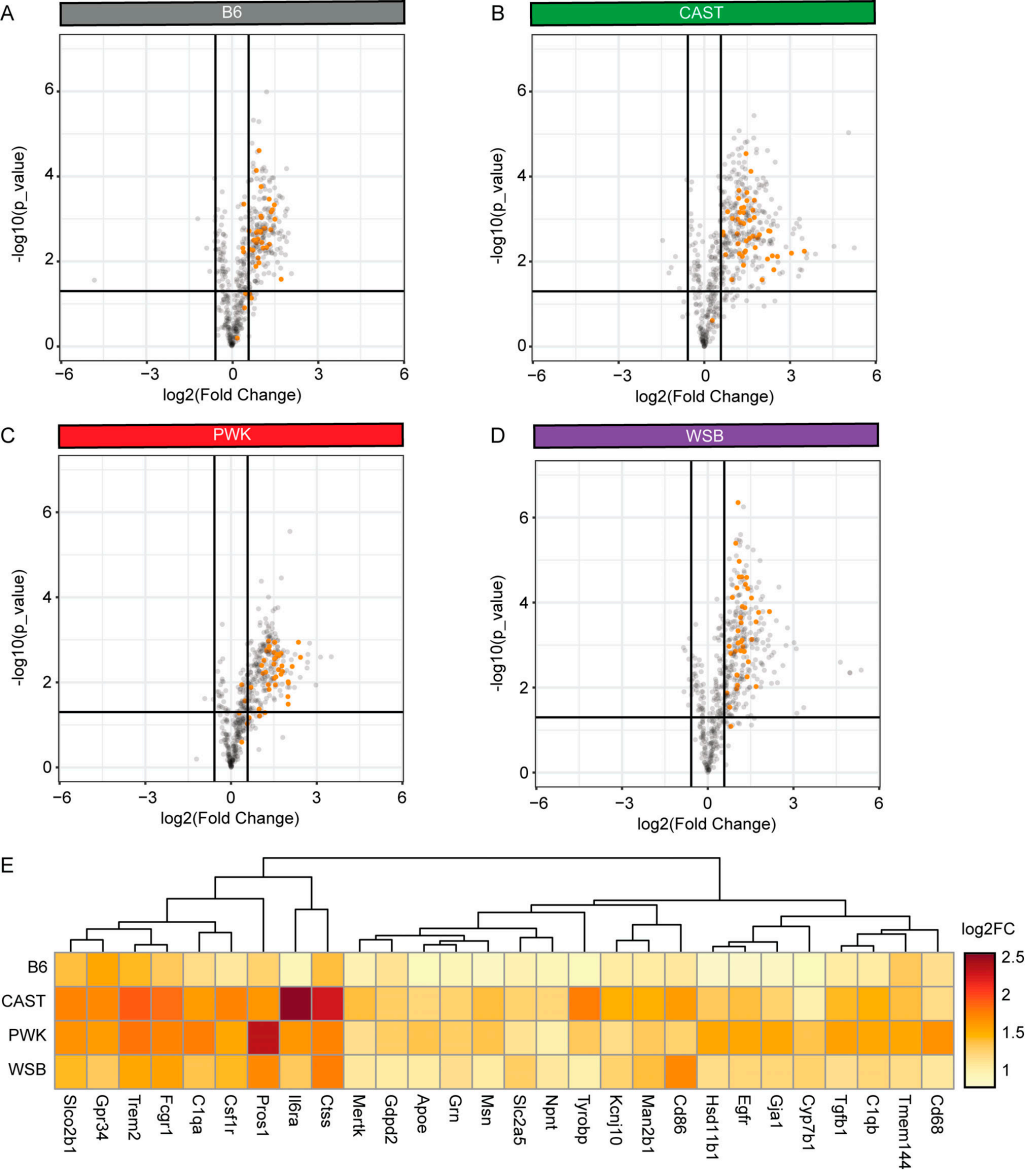
**Signature C: Darkorange module genes in shared inflammatory response to tau.** Volcano plot demonstrates the log_2_ fold change (x axis) and statistical significance (unadjusted P value, y axis) as measured via the nCounter mouse neuroinflammation panel. **(A–D)** (A) B6, (B) CAST, (C) PWK, and (D) WSB mice were analyzed separately to identify DEGs (|fold change| > 1.5, P < 0.05) in tau-injected mice compared to GFP-injected controls (*n* = 3/injection group, females only). Volcano plots are colored to highlight the 56 darkorange genes that are included on the mouse neuroinflammation panel. **(E)** Significant DEGs shared by all backgrounds are shown in a heatmap colored by log_2_FoldChange (FC) between hTauP301L-injected and eGFP-injected mice.

We found that 28 out of 56 darkorange module genes on the mouse neuroinflammation panel were significantly upregulated in AAV-hTauP301L–injected mice versus AAV-eGFP–injected controls across all genetic backgrounds, validating the involvement of these darkorange module genes in the immune response to tau (Fig. 7, A–D). Of these 28 genes, the highest fold change was observed in CAST and PWK mice (Fig. 7 E). Taken together, this suggests the genetic backgrounds that were most susceptible to tau seeding had the most increase in markers of inflammation.

### Signature C: Darkorange module and inflammatory response enriched in PWK and CAST

**Figure 8.**
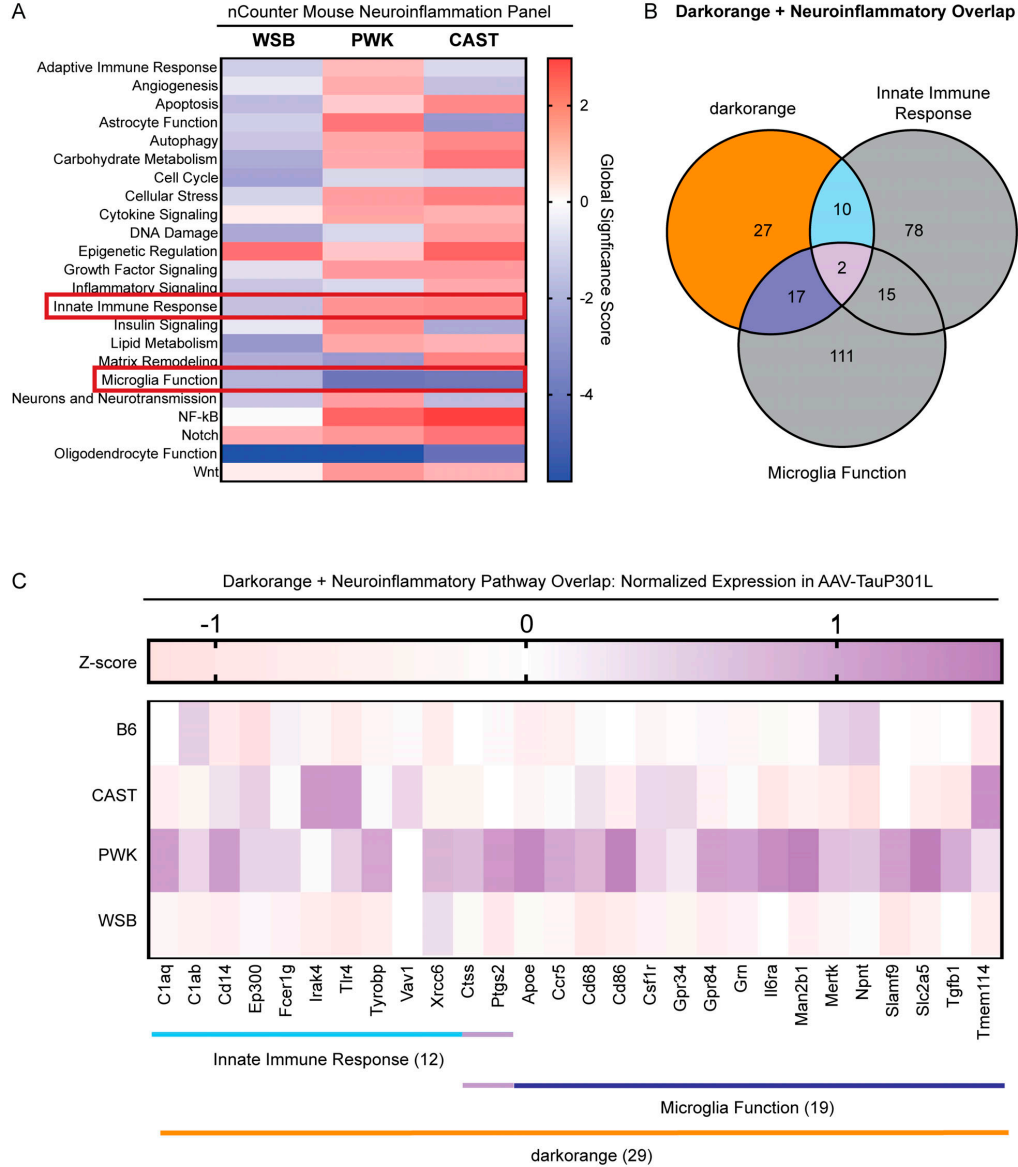
**Signature C: Darkorange module and inflammatory response are enriched in PWK and CAST.** DEGs were identified in CAST, PWK, and WSB mice injected with AAV-hTauP301L relative to B6.AAV-hTauP301L controls. **(A)** Global significance score for each nCounter mouse neuroinflammation annotation term was calculated using the Nanostring nSolver software. Red boxes indicate pathways (Innate Immune Response and Microglia Function) that were highlighted as they had the most drastic changes that were shared by both CAST and PWK mice. **(B)** An overlap of genes that make up the Signature C (darkorange module), Innate Immune Response, and Microglia Function. 29 genes were present in the darkorange module and at least one of the highlighted pathways. For the list of genes within these three pathways, please see Table S2 R. **(C)** Normalized expression of the 29 genes of interest within tau-injected mice was calculated as a z-score of normalized linear counts within tau-injected mice only. Heatmap of these 29 genes of interest shows that, within AAV-hTauP301L–injected mice, PWK mice had elevated expression of them.

In addition to identifying the shared inflammatory response across genetic backgrounds, we were also interested in determining which pathways were differentially enriched in wild-derived mice with respect to AAV-hTauP301L–injected B6 mice. Using the mouse neuroinflammation panel data, we performed pathway analysis with the Nanostring nSolver software (Fig. 8 A). Global significance score shows several pathways that were similarly modulated in PWK and CAST mice. This subset of pathways was of interest to us since PWK and CAST were the two genetic backgrounds susceptible to tau seeding (Fig. 2 G). The “Innate Immune Response” pathway was concordantly increased in PWK and CAST mice, while the “Microglia Function” pathway was decreased in both genetic backgrounds. The majority of darkorange module genes on the mouse neuroinflammation panel were annotated as being a part of the Innate Immune Response and/or Microglia Function (29 out of 56; Fig. 8 B and Table S2 R). Normalized expression of the 29 genes of interest within tau-injected mice was calculated as a z-score of normalized linear counts within tau-injected mice only. Strikingly, the normalized expression of these genes as measured by the nCounter platform identifies the largest effect in CAST and PWK mice compared to B6 and WSB mice (Fig. 8 C). In summary, these analyses of the targeted transcriptomic approach identified two microglial pathways and replicated our findings from bulk RNA-seq transcriptomics data.

### Resource: Gene-driven mouse selection and probing other datasets

**Figure 9.**
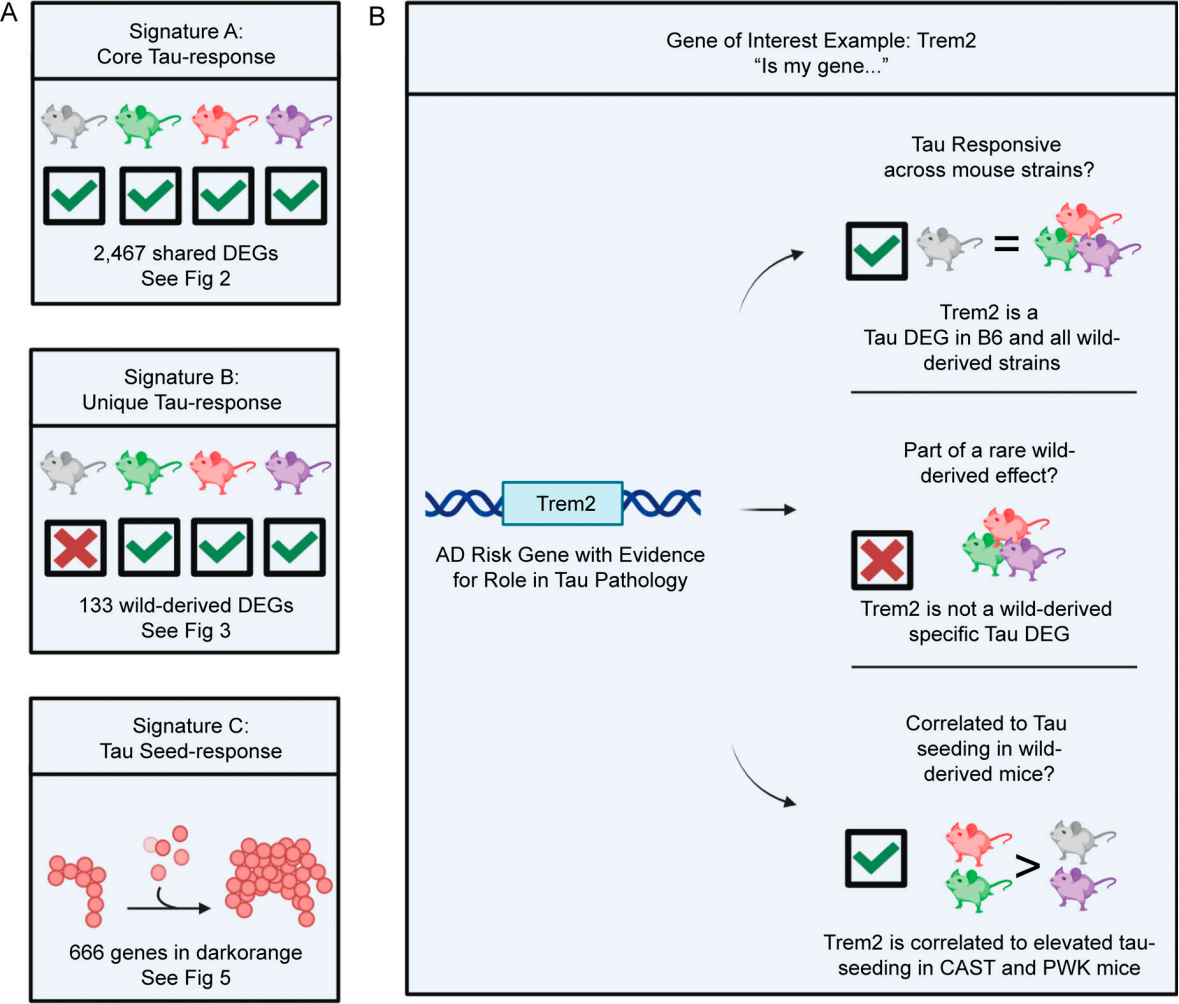
**Resource: Guideline to select a mouse genetic background to study Tau. (A)** Signatures A–C of this study represent a core response to expressing AAV-hTauP301L (Signature A), wild-derived specific response to AAV-hTauP301L expression (Signature B), and a tau seeding–associated module (Signature C). **(B)** Given a gene of interest, the resources in this paper can guide genetic background selection for functional studies in mice. For example, *Trem2*, a gene with strong evidence for a role in tau pathology, is present in Signature A (core Tau response) and Signature C (Tau seed response). Based on this evidence, while *Trem2* is differentially expressed in response to tau across all mouse strains, there is a possibility that it is involved in a CAST- or PWK-specific reaction to tau seeds.

The most direct use of this resource would be to take a gene-driven approach to mouse selection. Signatures A, B, and C are available as a resource to test whether a gene of interest is responsive across mouse strains, part of a rare wild-derived effect, or correlated to tau seeding in wild-derived mice (Fig. 9 A). For example, *Trem2*, a known AD risk gene expressed in microglia (Krasemann et al., 2017; Sims et al., 2017), is present in Signatures A and C. From this, we could conclude that *Trem2* is responsive to tau across mouse strains and is correlated to tau seeding observed in wild-derived mice (Fig. 9 B). This suggests that *Trem2* response to tau is resilient to changes in genetic background and contributes to the formation of tau seeds. This is one example of how this resource may be used to probe the effect of genetic background on a gene-by-gene basis.

**Figure S5.**
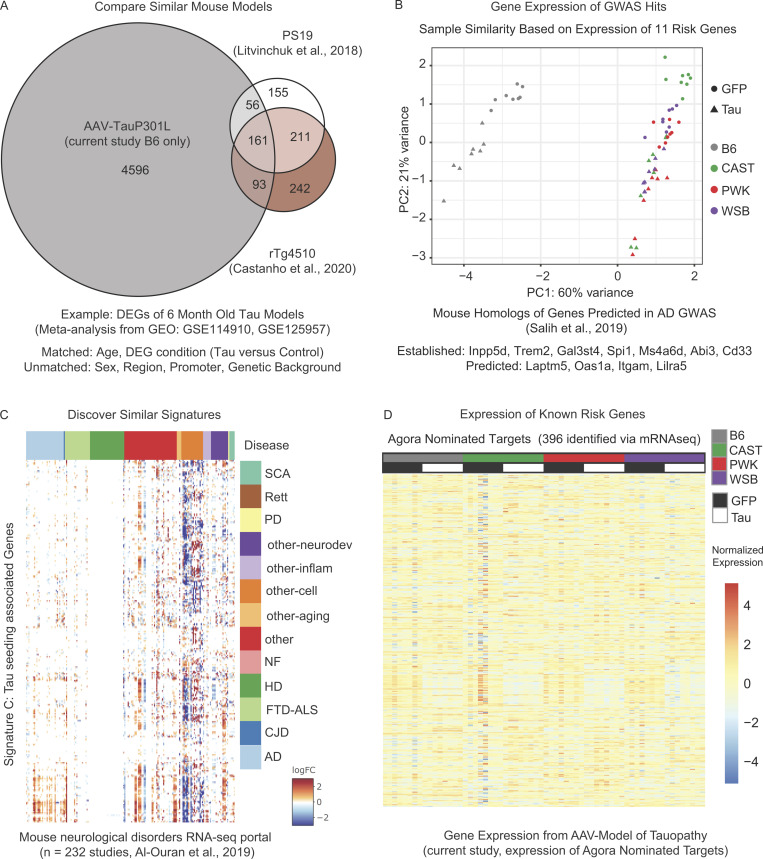
**Resource: Using transcriptional signatures to compare across studies. (A)** Analysis of publicly available data collected from mouse models of tauopathy (GSE114910, GSE125957) reveals a modest overlap between our AAV approach and similar tau models at 6 mo. Analysis was performed with models matched for age and DEG condition (Tau versus appropriate control). However, it was not possible to match for sex, exact region, the promoter used by the model, or the genetic background of the mice in each study. **(B)** Sample similarity was calculated via PCA of the expression of 11 mouse genes previously reported as mouse orthologs of human AD risk genes. Data suggests that these 11 genes were sufficient to group wild-derived mice separately from the classically inbred (B6) mice. **(C)** Analysis of the Signature C genes using the mouse neurological disorders RNA-seq portal. Portal includes studies in mouse models of spinocerebellar ataxia (SCA), Rett syndrome (Rett), Parkinson’s disease (PD), neurodevelopmental disorders (other-neurodev), inflammation and immunity (other-inflam), cell type specific expression (other-cell), aging (other-aging), neurofibromatosis (NF), Huntington’s disease (HD), frontotempral degeneration and amyotrophic lateral sclerosis (FTD-ALS), Creutzfeldt-Jakob disease (CJD), and AD. Genes in the Signature C are significantly upregulated in AD (light blue), downregulated in other cell types studies (orange), and largely absent within models of Huntington’s disease (dark green). **(D)** Heatmap of the Agora nominated target genes (*n* = 396) that were measured in our study via mRNA-seq.

To further demonstrate the usefulness of our resource, we also provide examples of how this dataset might be used in conjunction with other publicly available data (Fig. S5). First, this resource may be useful to compare viral models of tauopathy to traditional transgenic tau mouse models. Publicly available data were downloaded from similar mouse models of tauopathy (GSE114910, GSE125957) and DEGs identified in both datasets were compared with DEGs between B6.AAV-hTauP301L and B6.AAV-eGFP mice in our dataset. There is an overlap of 310 genes shared between our viral model and either of the two selected transgenic models (Fig. S5 A). This example meta-analysis suggests that there is a core response to TauP301L/S expression that is shared between these three models. This is another example of how this resource may be utilized in the study of tauopathy.

Finally, there is a wealth of data that already exists from human and mouse studies of neurodegeneration. Our resource may also be used in combination with any number of publicly available databases to drive future study. Here, we present three possible meta-analyses that can be done with our resources. First, a previous study identified 11 mouse orthologs of genes within human AD genome-wide association study (GWAS) loci that were co-expressed in mouse models of amyloid deposition (Salih et al., 2019). Using the expression of these 11 genes, wild-derived mice are transcriptionally distinct from B6 mice regardless of tau genotype (Fig. S5 B). Second, lists of genes of interest from this study could be searched for in external databases, like the mouse neurological disorders RNA-seq portal (Al-Ouran et al., 2019), to probe similar signatures across mouse models. Notably, the output of this portal showed a downregulation of Signature C genes within studies designated “other cell types” (Fig. S5 C). Third, the expression of ad hoc gene sets (i.e., the Agora nominated target list) could be analyzed to determine whether the expression of a known disease risk gene is altered by tau in a certain genetic background (Fig. 5 D). Taken together, these three approaches are examples of how one might use our resource in combination with publicly available datasets.

## Discussion

Recent studies in AD have shown that mouse genetic background can modulate Aβ accumulation (Onos et al., 2019), immune response (Yang et al., 2021), and tau propagation (Dujardin et al., 2022). To better understand how genetic background influences tauopathy, we aimed to create a resource of core- and unique-transcriptional signatures to tau expression based on mouse genetic background. Additionally, we wanted to determine if the seeding activity of tau was modified by genetic background. To better understand the pathogenicity of tau aggregates, it is important to investigate the initial seeding of tau and subsequent spreading/propagation, similar to studying Aβ in AD and α-synuclein in Parkinson’s disease (Peng et al., 2020). We report that the cortex of wild-derived CAST and PWK mice permits significantly higher prion-like proteopathic seeding activity of tau compared to that of B6 controls. To better understand the mechanisms involved, we performed a network analysis that implicated microglia in this strain-specific seeding activity and replicated our findings using a targeted neuroinflammation panel. Our data suggest that mouse genetic background is an important factor when studying immune responses to pathological tau species.

For this study, we selected three wild-derived genetic backgrounds (CAST, PWK, and WSB) to compare with B6. Using a genotyping array, gigaMUGA, we report that these three wild-derived strains contain many variants in the nominated targets from the AMP-AD database (Fig. 1). A comprehensive list of all variants in these wild-derived mice and 85 other strains can be found in the Jackson Laboratory’s Mouse Genome Database (Blake et al., 2021). Transcriptomic data from Onos and colleagues showed a compelling effect of these three wild-derived strains on amyloid accumulation (Onos et al., 2019; Yang et al., 2021). To compare the response to the two hallmarks of AD, amyloid, and tau, we decided to investigate these same mouse strains. Our data suggest that these wild-derived strains are an ideal resource for investigating the contribution of genetic variation to the study of AD and other tauopathies.

With AD mouse models, transcriptional signatures have been an important experimental readout. Several studies have shown that this hypothesis-generating, unbiased readout can be used to investigate Aβ accumulation (Sierksma et al., 2020), region-specific expression of tau (Castanho et al., 2020), and activated immune response (Kang et al., 2018). Previously, genetic background has been shown to influence the amount of tau and the presence of at least one phospho-epitope in tau transgenic models (Eskandari-Sedighi et al., 2017; Yanagisawa et al., 2021; Bailey et al., 2014). However, no transcriptional information or mechanism of action has ever been proposed. To test the effect of wild-derived genetic background and generate hypotheses about the responsible mechanisms, we used transcriptional signatures as our main readout. Importantly, our study shows that the core transcriptional response to tau across different genetic backgrounds is enriched for pathways of neurodegeneration (Fig. 3). This finding suggests that the fundamental pathways involved in studying mouse models of dementia do not change across genetic backgrounds. As preclinical studies continue to make direct comparisons between human and mouse transcriptomics (Monzón-Sandoval et al., 2022; Onos et al., 2022), our list of core genes can be interpreted as robust tau-responsive genes for future study.

However, we argue that it is just as important to understand what transcriptomic response is modulated by different genetic backgrounds. Previous studies of Trem2 on mixed background mice indicated an allele inherited by the SJL strain that unknowingly introduced a missense mutation (Yang et al., 2021). In previous studies, we have addressed this issue by excluding mice that are homozygous for the SJL allele from analysis (Karahan et al., 2021). However, for investigators planning to study novel risk genes, it would be impossible to control for every naturally occurring variant. We propose using these differences, specifically in the study of tauopathy, to our advantage. Should researchers consider focusing on any gene of interest, it is critical that we first understand what aspects of mouse biology are causing the genetic background to modulate tau phenotypes. We report a unique or “segregating” response to tau in wild-derived mice (Fig. 4). For example, WSB mice appear to have a unique downregulation in two genes involved in motor transport, *Kif14* and *Myl1* (Fig. 3 D). For those studying the role of motor transport in AD (Gan et al., 2020), the WSB background may provide insights that would not be observed using B6 mice. Other conclusions from the wild-derived unique responses could explain unexpected negative data when using B6 mice. This would be one of the barriers to translating research findings into humans.

Lastly, to understand if genetic background modulates the pathogenicity of tau, we investigated the effect of mouse strain difference on the tau seeding activity. We found an increase in tau seeding activity in the cortex of CAST and PWK mice (Fig. 2). More research is necessary to understand which tau species contribute to the in-seeding activity in the cortex of wild-derived mice. It is possible that modifiers or interactors of tau exist in CAST and PWK mice. Our previous research has demonstrated that interactors like Bassoon (*Bsn*) contribute to the ability of tau to seed and induce neurotoxicity (Martinez et al., 2022). As a pathological readout, seeding activity has been shown to identify the action of high molecular weight tau (Martinez et al., 2022) and even differentiate between specific conformers in 3R/4R tau diseases (Kraus et al., 2019). Importantly, our network analysis identified a module of genes associated with this increase of tau seeding activity in CAST and PWK mice (Fig. 5). Our enrichment analysis and comparison to previously published microglial contextual states suggest the importance of microglia in tau seeding (Fig. 6). Interestingly, we see a considerable overlap when comparing the tau seeding–associated genes in wild-derived mouse strains to a previous study of amyloid response in wild-derived strains (Onos et al., 2019). These include *Trem2*, *Tyrobp*, *Tgfbr2*, and *Cd68*.

To independently validate the module discovered via network analysis of whole transcriptomic data (Figs. 5 and 6), we selected a targeted assay (Nanostring Neuroinflammation Panel) that provides a comprehensive survey of neuroinflammation (Fig. 7). Analysis of the effect of genetic background on hTauP301L expressing mice further supported the role of Signature C (darkorange module) in the innate immune response and microglial function (Fig. 8). By combining unbiased whole transcriptomic analyses with targeted RNA probing, we have identified this subset of genes involved with tau seeding and increased inflammatory response. Future experiments are warranted to test the functional effects of these genes by knocking down one by one, in combination and in a cell-type-specific manner.

In light of the increasing effort to identify disease- and context-specific glial states (Keren-Shaul et al., 2017; Paolicelli et al., 2022; Ezerskiy et al., 2022), single-cell RNA-seq will be necessary to better understand which microglia cell types are involved in this phenotype. However, we believe that this study provides compelling evidence that microglia within PWK and CAST mice contribute to an increased susceptibility to tau seeding. Future studies would be necessary to understand what mechanisms contribute to this differential susceptibility across strains. Additionally, this differential susceptibility of PWK and CAST mice to tauopathy implicates them as promising founder strains in future genetic mapping studies.

In conclusion, we determined strain-specific genetic variants in wild-derived mouse genetic background using Illumina Infinium platform and reported three transcriptional signatures identified via RNA-seq. First, a core tau-responsive transcriptional signature that is not affected by genetic background (Signature A). Second, a unique transcriptional response to tau that may indicate wild-derived mice should be used to study certain risk genes (Signature B). Third, a tau seeding activity–associated transcriptional signature that implicates microglia (Signature C). Our data provide a resource for investigating tau in mouse models of AD and other tauopathies (Fig. 9 and Fig. S5). Given that most therapeutic approaches are tested in mice before progressing to clinical trials, including wild-derived mice may enhance the translatability to treating patients with different genetic backgrounds.

## Materials and methods

### Mouse strains and genotyping

This study was designed to investigate the role of genetic background in the pathogenesis of tauopathy. To achieve this, we purchased breeders from genetically diverse mouse strains from the Jackson Laboratory (C57BL6/J: Stock #000664 RRID:IMSR_JAX:000664, CAST/EiJ: Stock #000928 RRID:IMSR_JAX:000928, PWK/PhJ: Stock #003715 RRID:IMSR_JAX:003715, and WSB/EiJ: Stock #001145 RRID:IMSR_JAX:001145). All procedures and animal work were approved by the Indiana University School of Medicine Institutional Animal Care and Use Committee (Protocol 21149).

Tail samples from a pilot cohort were collected and sent to GeneSeek (Neogen) for genotyping on the gigaMUGA (Mouse Universal Genotyping Array) platform. This array contains 143,259 SNP and copy number variants markers that were selected to be informative in wild-derived mice and multiple *Mus* species (Morgan et al., 2015). The results published in this study are in part based on data obtained from Agora (https://agora.adknowledgeportal.org/), a platform initially developed by the National Institute on Aging–funded AMP-AD consortium that shares evidence in support of AD target discovery. In argyle (Morgan, 2015), variants were recoded and filtered based on the position of the AMP-AD nominated target genes (accessed March 1, 2021). Variants were then visualized across the whole mouse genome using rCircos (v1.2.2) and at the Inpp5d locus using GeneVisR (v3.17).

### Intracerebroventricular injections of AAV

To model tau aggregation, we injected mice of each genetic background with either AAV-hTauP301L (AAV9-CBA/CMV-hTauP301L-WPRE-polyA) or AAV-eGFP (AAV9-CBA/CMV-eGFP-WPRE-polyA). Sample size was determined by the pilot study described in the Results section. The final sample size reached our requirement based on power analysis (B6 = 20, CAST = 20, PWK = 19, WSB = 24). We selected AAV9, which has been shown to have high intracellular expression without an effect on mouse genetic background (He et al., 2019).

A full protocol for this approach was previously published (Kim et al., 2014; Passini et al., 2003). In brief, breeder cages were checked three times daily to ensure injection occurred between 12 and 24 h after birth (Postnatal Day 0, P0). P0 mice were cryo-anesthetized for 8 min on ice. Using a 32-gauge needle, 2-mm-deep injections were made into each lateral ventricle (0.8–1 mm later from the sagittal suture and hallway between lambda and bregma) at a 45° angle. A total of 2 μl of virus was injected per ventricle (4 × 10^10^ viral particles/mouse) to express each construct. The injection was performed slowly and the needle was held in place for an additional 30 s. Upon removal of the needle, if more than 0.2 μl of the virus leaks out of the injection site, the animal is immediately euthanized. Surviving mice were placed on a warming pad until the pups began to move and were promptly returned to their parent cage.

### Tissue harvesting and sample preparation

At 6 mo of age (182.1 ±4.9 d, mean ± standard deviation), mice were anesthetized using carbon dioxide for 2.5 min. Brains were promptly removed and the left hemisphere was fixed in 4% paraformaldehyde for 24 h at 4°C to be stored for histology. Tissue samples were embedded in paraffin and sectioned at Histology Lab Service Core at the Indiana Center for Musculoskeletal Health. 5-µm-thick coronal sections (at bregma −1.46, −1.94, and −2.46 mm) were transferred to charged microscope slides and stored at room temperature. The anterior cortex, posterior cortex, hippocampus, and cerebellum were dissected from the right hemisphere and flash-frozen with liquid nitrogen. Samples were stored at −80°C.

### Protein preparation

Samples were weighed and prepared in 1X Tris-buffered saline (TBS) at 100 mg of tissue per milliliter of lysate. After a brief gentle mechanical dissociation, samples were aliquoted for either RNA or protein extraction. The aliquot designated for protein extraction was homogenized via sonication and centrifuged at maximum speed for 15 min at 4°C. The supernatant, referred to as “TBS-soluble,” was then normalized to 2.0 mg/ml via bicinchoninic acid assay (Thermo Fisher Scientific) and stored at −80°C until analyzed.

### Tau seeding assay

The seeding assay was performed using TauRD P301S FRET Biosensor Cells (Holmes et al., 2014). In brief, we obtained HEK293-T cells expressing truncated TauP301S containing only the RD fused to either CFP or YFP (ATCC CRL-3275; RRID:CVCL_DA04). These biosensor cells were plated in a 96-well plate at 30,000 cells per well and incubated at 37°C overnight. After 24 h, cells were transfected with 20 μg of TBS-soluble brain lysate using Lipofectamine 2000. Total brain lysate was transfected without normalizing to total tau levels. After an additional 48 h at 37°C, cells were harvested and FRET+ signal was measured via Flow Cytometry (BD LSRFortessa X-20 with High Throughput Sampler). Data analysis was performed in FlowJo (v10.0, RRID:SCR_008520). Our gating strategy for singlet selection, CFP background removal, and FRET+ signal (BV510 channel) were performed as previously described (Martinez et al., 2022). Tau seeding activity was quantified as the percent of total cells with FRET+ signal.

### Western blotting

A total of 20 μg of protein was loaded onto a 4–20% TGX gel (Bio-Rad), separated by gel electrophoresis, and transferred onto nitrocellulose membranes. Membranes were blocked with 5% milk in TBS containing 0.05% Tween20. Blots were probed with polyclonal rabbit anti-human tau (1:1,000; RRID:AB_10013724; DAKO), anti-phospho tau Th231 (1:1,000, MN1040 RRID:AB_223649; Invitrogen), and Vinculin (1:1,000, V9131-100UL RRID:AB_477629; Sigma-Aldrich). Antibodies were incubated overnight at 4°C. The next day, membranes were washed with TBS containing 0.05% Tween20 and incubated with anti-mouse or anti-rabbit IgG antibodies based on the host of the primary antibody. Membranes were developed by chemiluminescence (SuperSignal West Pico, 34577; Thermo Fisher Scientific).

### Tau protein quantification from mouse cortex

Quantification of total tau protein and phosphor-tauThr231 were measured using a kit from Meso Scale Diagnostics (K15121D). To read both total and phosphorylated tau simultaneously, normalized protein lysate from brain cortex (2.0 mg/ml) was diluted 1:2,000. Signal detection was performed via MESO QuickPlex SQ 120 MM and analysis was done using Methodical Mind software (Meso Scale Diagnostics).

### Histology and immunohistochemistry

Slides containing mounted coronal sections were deparaffinized using xylene. Antigen retrieval was performed with High pH IHC antigen retrieval solution (00-4956-58; Invitrogen) for 10 min in a microwave oven.

For 3,3′-diaminobenzidine (DAB) staining, endogenous peroxidation was quenched by incubating slides in a solution containing 10% methanol and 3% hydrogen peroxide in phosphate-buffed saline (PBS) for 10 min. Slides were blocked with 5% normal goat serum in PBS containing 0.01% Triton X-100. Sections were incubated with primary antibodies overnight at 4°C. Human tau-specific (HT7, 1:200; MN1000 RRID:AB_2314654; Invitrogen) antibody was diluted in 2.5% normal goat serum in PBS containing 0.01% Triton X-100. Sections were washed and incubated with biotinylated goat anti-mouse secondary antibody (1:100, RRID:AB_228305; Thermo Fisher Scientific) at room temperature for 1 h. Antibody detection for DAB development was done using the Vectastain ABC Elite (PK6100; Vector Laboratories) and DAB Peroxidase Substrate kits (SK-4100; Vector Laboratories). When applicable, hematoxylin and bluing staining were performed by incubating 5 min with hematoxylin (H-3502; Vector Laboratories), then rinsed twice in distilled water and adding the bluing reagent for 15 s (H-3502; Vector Laboratories). Slides were washed three times in ethanol 100%. Sections were dehydrated and cleared with ethanol and xylene and immediately coverslipped with mounting medium (1900333; Epredia).

### RNA preparation for transcriptomic analyses

Total RNA was extracted from posterior cortex brain tissue using TRIzol (MRC). RNA concentration and quality were determined via Nanodrop 200 Spectrophotometer.

For real-time quantitative polymerase chain reaction (qPCR), cDNA was prepared using a high-capacity cDNA reverse transcription kit (Applied Biosystems). qPCR was performed in QuantStudio 3 using the recommended protocol for FAST SYBR (Applied Biosystems) with the following primers: human specific *MAPT* forward 5′-TTG​CTC​AGG​TCA​ACT​GGT​TT-3′, human specific *MAPT* reverse 5′-ACT​GAG​AAC​CTG​AAG​CAC​CA-3′, mouse *Gapdh* forward 5′-AAG​GTG​AAG​GTC​GGA​GTC​AAC-3′, mouse *Gapdh* reverse 5′-GGG​GTC​ATT​GAT​GGC​AAC​AAT​A-3′. Relative mRNA levels were calculated by comparative cycle threshold (ΔΔCt).

For mRNA-seq, total RNA was concentrated and purified using RNA Clean-Up & Concentrator-5 kit (Zymo Research). RNA integrity number and concentration were determined via TapeStation RNA tape (Agilent). Sequencing was performed by the Center for Medical Genomics at the Indiana University School of Medicine (Indianapolis, IN). Libraries were created from 100 ng of total RNA using mRNA HyperPrep kit (KAPA). Libraries were then checked for quality and loaded at a concentration of 300 pM on a flow cell for 100 bp paired-end sequencing (S4_200cycle flow cell v1.5). Sequencing was then performed on an Illumina NovaSeq 6000 at an average sequencing depth of ∼30 million reads per sample.

For Nanostring, total RNA from the cortex of female mice injected with either AAV-hTauP301L (*n* = 3/genetic background) or AAV-eGFP (*n* = 3/genetic background) was used. Targeted measurement of the 757 genes on the mouse neuroinflammation gene expression panel was performed on the nCounter platform (Nanostring) per the manufacturer’s protocol.

### Transcriptomic analyses

Reads were mapped to the respective reference genome of each genetic background (B6-UCSC/refGene mm10, CAST-GCA_001624445.1, PWK-GCA_001624775.1, and WSB-GCA_001624835.1) using RNA-seq aligner STAR (v.2.7.10a). See supplement information for sequencing and mapping statistics (Table S2, M–Q). Reads were assigned to genomic features using featureCounts (Liao et al., 2014). Raw read counts were analyzed for either differential expression analysis in DESeq2 (Love et al., 2014; v1.36.0; RRID:SCR_000154) or network analysis using WGCNA (Langfelder and Horvath, 2008; v1.71; RRID:SCR_003302).

For differential expression analysis, two separate strategies were applied. To identify a core transcriptional response to expressing hTauP301L, analysis was first done on each genetic background separately. This allowed for strain-specific genes that are not annotated in the mm10 reference genome to be included in our initial analyses. Within each genetic background, genes with a raw read count of <10 were filtered out. Differential gene expression was then calculated for AAV-hTauP301L–injected mice relative to AAV-eGFP control (B6 = 17,963, CAST = 22,426, PWK = 22,100, WSB = 22,399 genes after filtering). Up- and downregulated genes were defined using a significance cutoff of 0.05 (Benjamini Hochberg adjusted P values) and a 1.5-fold change (after *apeglm* effect size shrinkage [Zhu et al., 2019]).

The second differential expression analysis aimed to find unique responses to hTauP301L without the effect of genetic background or tau expression alone. To do this, raw read counts across genetic backgrounds were merged keeping only genes that were annotated in the mouse reference genome (mm10). After merging, 19,468 genes were identified at least once in each genetic background and 17,240 had at least 10 read counts across all genetic backgrounds. These 17,240 genes were used in all downstream analyses (PCA, unique transcriptional response, WGCNA). Note that there were several genes mapped to wild-derived backgrounds that were removed from the analysis because they correspond to more than one gene on the reference genome (CAST = 12, PWK = 12, WSB = 14 multimapped genes removed). These were all either predicted genes (“Gm” prefix) with the exception of one small nucleolar RNA (Snora43).

Raw reads underwent variance stabilization transformation and PCA was used to identify potential outliers. Differential gene expression was then performed with genetic background as an interaction (∼Injection+GeneticBackground+Injection:GeneticBackground). The goal of this calculation is to identify tau-responsive genes that were dependent solely on genetic background. Up- and downregulated genes were defined using a significance cutoff of 0.05 (Benjamini Hochberg adjusted P values) and a 1.5-fold change (after *apeglm* effect size shrinkage) for each interaction term (Tx_Tau_CAST, Tx_Tau_PWK, and Tx_Tau_WSB) with B6 as the baseline.

We then performed WGCNA to identify modules of co-expressed genes that could explain the variation we reported in tau seeding activity across genetic backgrounds. For this analysis, we returned to the raw count matrices without any normalization or filtering as recommended by the authors of the pipeline (Langfelder and Horvath, 2008). After outlier removal in WGCNA, we were left with a total of 60 samples (Fig. S4 A). Our data did not reach the suggested scale-free topology model fit cutoff of 0.9 (Fig. S4 B). A soft power threshold of six was selected based on the suggestions by the authors of the pipeline for a dataset with more than 40 samples. A total of 60 modules were identified (Fig. S4 C) and 21 of them were significantly associated with FRET+ seeding activity or wild-derived genetic background (Fig. S4 D; Pearson’s correlation, P < 0.05).

For comparing to similar mouse models of tauopathy (PS19: GSE114910 and rTg4510: GSE125957), data were accessed on Gene Expression Omnibus (GEO; RRID:SCR_005012) and analyzed in DESeq2 as described for our own data. DEG analysis was done on each study separately relative to the controls described in the original studies (Castanho et al., 2020; Litvinchuk et al., 2018).

### Multi-dimensional module identification

To prioritize modules discovered by WGCNA, the module–trait relationship was visualized in two dimensions (x axis: association to wild-derived genetic background, y axis: association to seeding activity). Modules were sized according to the number of genes with each and the opacity set based on whether it was significant in both dimensions (black), one dimension (opacity 50%), or neither (opacity 10%). Gene significance for each of the four modules was calculated as described by the authors of the WGCNA package.

### Mediation analysis

First, high-dimensional mediation analysis (Zhang et al., 2016) was performed using R Package for High-Dimensional Mediation Analysis (http://github.com/dclarkboucher/hdmed; Clark-Boucher et al., 2023 *Preprint*). Alpha, beta, and P values were calculated using the default parameters of the mediate_hima() function where A = genetic background, M = matrix containing the eigengene value of each WGCNA module, and Y = tau seeding measured by FRET.

Second, we performed a Bayesian module selection approach to test whether darkorange was acting as a complete or partial mediator (Crouse et al., 2022). Using the default effect size priors and the complete model options of the bmediatR package, we calculated the posterior model probability for complete mediation, partial mediation, and other non-mediation.

### Nanostring analysis

Nanostring gene expression analysis was first performed in the nSolver software (RRID:SCR_003420) to compare the expression of hTauP301L versus eGFP controls in each genetic background separately. Then, to investigate the role of wild-derived genetic background, hTauP301L-injected wild-derived mice were compared with B6.hTauP301L controls (i.e., PWK.hTauP301L versus B6.hTauP301L). Global significance scores against the Nanostring annotation pathways were calculated using the nSolver software. To compare the gene expression levels between the genotypes, we performed a Z-score transformation for each gene identified in the Innate Immune Response and Microglia Function that overlap with the darkorange module.

### Signatures from literature for comparison

To compare gene signatures identified in our study to those in the literature, we mined several publicly available datasets.

First, Onos and colleagues reported a list of genes associated with a wild-derived specific response to amyloid transgene (Onos et al., 2019). This list of 35 genes was identified as the “light yellow” module via WGCNA module–trait analyses for genetic background and presence of the APP/PS1 transgene (Onos et al., 2019).

To infer the microglial state, we selected known markers determined by previously published single-cell RNA-seq studies for DAM (Keren-Shaul et al., 2017) and homeostatic microglia (Li et al., 2019). Marker genes were defined using those passing P < 0.05 (Bonferonni corrected) and greater than positive 1.5-fold change. From these lists of genes, we then removed any ribosomal genes and any unannotated genes from either the Riken mouse genome encyclopedia (Hayashizaki, 2003) or Mouse Genome Informatics (Blake et al., 2021; prefixes “Gm-” or suffixes “-Rik”). Using this approach, we identified 170 DAM markers and 175 homeostatic markers.

To characterize whether genes at known AD risk loci were responsible for the effect of genetic background, we selected 11 mouse genes previously reported as orthologs of genes within human AD GWAS loci (Salih et al., 2019).

We demonstrated the ability to use our gene lists with external databases like the mouse neurological disorders RNA-seq portal (Al-Ouran et al., 2019) to probe similar signatures across mouse models. Output from this database was unaltered and can be accessed by inputting the Signature C gene list into the publicly available portal.

### Enrichment and scoring analyses

Enrichment analyses for core tau signature (Table S2 J), unique tau signature (Table S2 K), and WGCNA modules (Table S2 L) were performed in gProfiler2 (R Client, v0.2.1). Output includes enrichment for Gene Ontology terms, Reactome, TRANSFAC, miRTarBase, Human Protein Atlas, Comprehensive Resource of Mammalian Protein Complexes, Human Phenotype Ontology, and WikiPathways. For enrichment of our signatures against other published datasets, we performed a Fisher’s exact test using the stats package in R (v4.2.1). Given the size of test signature (A), the size of the signature from literature (B), the size of overlap between signature A/B (t), and background (*n* = whole transcriptome), enrichment was considered significant if P < 0.05. The command stats::dhyper(t:B,A,n-A,B) returned the P value for Fisher’s exact enrichment.

PCA was utilized to score the expression of lists of genes. Given a list of genes of interest, either from previous studies or our own, raw reads underwent variance stabilization transformation and PCA was performed using the plotPCA() function in DESeq2. We then plotted the first (PC1) to summarize the expression of those genes of interest.

### Statistical analysis and figure creation

For analysis of tau pathology, analysis was done via one-way analysis of variance (ANOVA) followed by Tukey honest significant difference post-hoc test. Statistical tests are reported in the figure legends with sample size, F statistic, degrees of freedom, and P value. Where appropriate, figures are labeled with the exact P value (P > 0.05), *(P < 0.05), **(P < 0.01), and ***(P < 0.001). All analysis was done in R (v4.2.1) and figures were created using ggplot2 (v3.3.6; RRID:SCR_014601), Cytoscape (v3.9.1; RRID:SCR_003032), pheatmap (v1.0.12), and BioRender.com (RRID:SCR_018361).

### Online supplemental material

Supplemental figures include a description of a pilot study to determine sample size (Fig. S1), specific expression of human tau (HT7) in AAV-hTauP301L compared to AAV-eGFP injected control (Fig. S2), a summary of genetic background–specific transcriptomic analyses not shown in the main figures (Fig. S3), a summary of WGCNA analysis (Fig. S4), and expanded suggestions on how to use our resource (Fig. S5). Supplemental files contain tables with wild-derived AMP-AD variant information (Table S1) and summaries of transcriptomic analyses (Table S2).

## Supplementary Material

Table S1 shows genotyped variants of wild-derived mice in AMP-AD nominated genes.

Table S2 is a summary of genetic background–specific transcriptomic analyses.

SourceData F2 is the source file for Fig. 2.

## Data Availability

The data underlying Fig. 1 are available in the published article and its online supplemental material. The data underlying mRNA-seq experiments in Figs. 2, 3, 4, 5, and 6 are openly available in GEO at GSE223840. The data underlying Nanostring experiments in Figs. 7 and 8 are openly available in GEO at GSE233988. All remaining data can be found in the supplemental information in this manuscript.
