# Supplementary figures and images for: Network analysis identifies strain-dependent response to tau and tau seeding-associated genes
Source: J Exp Med. 2023 Aug 22;220(11):e20230180. doi: 10.1084/jem.20230180 (PMC10443211; doi:10.1084/jem.20230180)

pTau  
Thr231

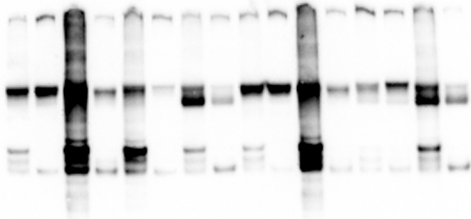

Total Tau

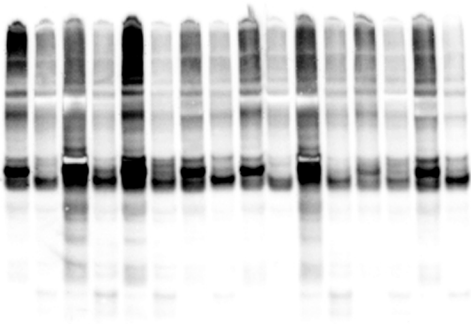

Vinculin

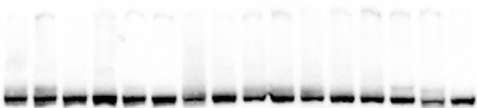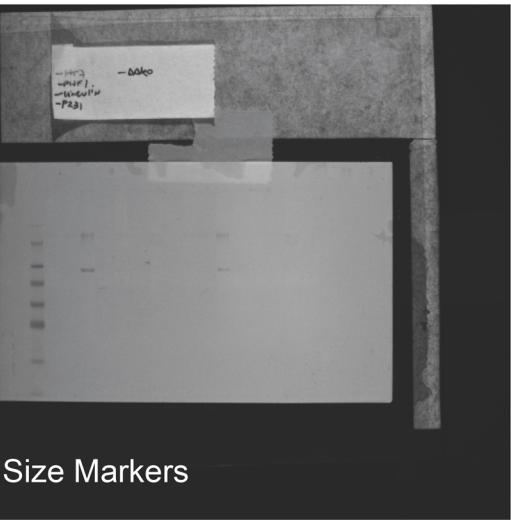

Corresponding Figure 2B

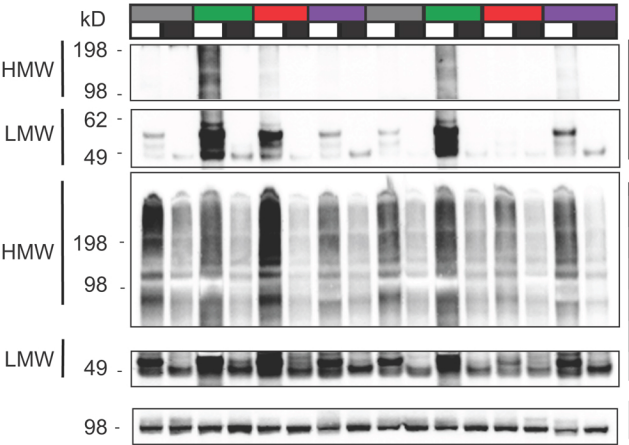

Supplement: SourceData F2 — is the source file for Fig. 2. [file JEM_20230180_SourceDataF2.pdf]
